# *Helicobacter pylori* binds human Annexins via Lipopolysaccharide to interfere with Toll-like Receptor 4 signaling

**DOI:** 10.1371/journal.ppat.1010326

**Published:** 2022-02-17

**Authors:** Barbara Schmidinger, Kristina Petri, Clara Lettl, Hong Li, Sukumar Namineni, Hellen Ishikawa-Ankerhold, Luisa Fernanda Jiménez-Soto, Rainer Haas

**Affiliations:** 1 Chair of Medical Microbiology and Hospital Epidemiology, Max von Pettenkofer Institute, Faculty of Medicine, LMU Munich, Germany; 2 West China Marshall Research Center for Infectious Diseases, Center of Infectious Diseases, Division of Infectious Diseases, State Key Laboratory of Biotherapy, West China Hospital, Sichuan University, Chengdu, China; 3 Department of Internal Medicine I, Faculty of Medicine, LMU Munich, Germany; 4 Walter Brendel Centre of Experimental Medicine, University Hospital, LMU Munich, Germany; 5 German Center for Infection Research (DZIF), LMU Munich, Germany; University of Illinois, UNITED STATES

## Abstract

*Helicobacter pylori* colonizes half of the global population and causes gastritis, peptic ulcer disease or gastric cancer. In this study, we were interested in human annexin (ANX), which comprises a protein family with diverse and partly unknown physiological functions, but with a potential role in microbial infections and possible involvement in gastric cancer. We demonstrate here for the first time that *H*. *pylori* is able to specifically bind ANXs. Binding studies with purified *H*. *pylori* LPS and specific *H*. *pylori* LPS mutant strains indicated binding of ANXA5 to lipid A, which was dependent on the lipid A phosphorylation status. Remarkably, ANXA5 binding almost completely inhibited LPS-mediated Toll-like receptor 4- (TLR4) signaling in a TLR4-specific reporter cell line. Furthermore, the interaction is relevant for gastric colonization, as a mouse-adapted *H*. *pylori* increased its ANXA5 binding capacity after gastric passage and its ANXA5 incubation *in vitro* interfered with TLR4 signaling. Moreover, both ANXA2 and ANXA5 levels were upregulated in *H*. *pylori*-infected human gastric tissue, and *H*. *pylori* can be found in close association with ANXs in the human stomach. Furthermore, an inhibitory effect of ANXA5 binding for CagA translocation could be confirmed. Taken together, our results highlight an adaptive ability of *H*. *pylori* to interact with the host cell factor ANX potentially dampening innate immune recognition.

## Author summary

*H*. *pylori* is very well adapted to its natural habitat, the human gastric mucosa. For this purpose, the bacterium has evolved a number of highly specific virulence factors, such as the *cag-*type IV secretion system, vacuolating cytotoxin A (VacA) or secreted gamma-glutamyl transpeptidase. An important function of these bacterial factors is to manipulate the host immune response to enable a chronic *H*. *pylori* infection. The present work identifies a new player in this process. Here, we have discovered that *H*. *pylori*, as well as several other bacterial species, can bind human annexins (ANX), suggesting a more widespread phenomenon. We show that *H*. *pylori* specifically binds ANXA5 via lipid A. The interaction is strictly dependent on calcium and modulated by the phosphorylation status of lipid A. Notably, ANXA5 binding strongly inhibits LPS-mediated Toll-like receptor 4 (TLR4) signal transduction, suggesting that *H*. *pylori* exploits ANXs binding to avoid its recognition by this important receptor of the innate immune system. The study thus provides novel molecular and mechanistic insights into a further strategy of *H*. *pylori* to successfully evade recognition by the host.

## Introduction

Infection with *Helicobacter pylori* (*H*. *pylori*) is responsible for chronic gastritis, gastroduodenal ulcers, and is also a high risk factor for the development of mucosa-associated lymphoid tissue (MALT) lymphoma as well as gastric adenocarcinoma [1]. Two major virulence factors of *H*. *pylori* are the active vacuolating toxin VacA [2] and a 40kb pathogenicity island (*cag*-PAI) [3] encoding the *cag*-type IV secretion system *(cag*-T4SS). The *cag*-T4SS is used by *H*. *pylori* to inject the 120–145 kDa immunodominant protein CagA into the host cell [4]. The presence of a functional *cag*-PAI correlates with the development of severe gastric inflammation, gastric or duodenal ulcer and gastric cancer [5].

The membranes of the *H*. *pylori* cell envelope consist of simple lipids (such as free fatty acids, triglycerides and cholesterol) and phospholipids [6] and harbor a set of outer membrane proteins acting as porins or specific adhesins [7]. The fatty acid composition of *H*. *pylori* is uncommon and distinct from the closely related *Campylobacter jejuni* (*C*. *jejuni*) [8]. As *H*. *pylori* is not able to synthesize cholesterol itself the bacteria extract cholesterol from the host cell and integrate it into their membrane after it has been α-glycosylated by its cholesterol-α-glucosyltransferase (Cgt) [9]. Moreover, the lipopolysaccharide (LPS) of *H*. *pylori* is exceptional in many ways. It is appropriately adapted to evade the host immune system and exhibits low endotoxicity compared to the LPS of other pathogens. Therefore, it is considered as an important virulence factor in the persistent colonization of the human stomach [10]. The O-antigen side chains of *H*. *pylori* LPS are modified in many ways to resemble Lewis blood group antigens and therefore serve as molecular mimicry [11]. Due to modifications in the phosphorylation and acylation pattern (underphosphorylation and–acylation) lipid A of *H*. *pylori* is less negatively charged, and therefore more resistant to cationic antimicrobial peptides (CAMPs) and less biologically active [10, 12]. Lipid A can also be modified by a phosphoethanolamine transferase and consists of longer fatty acid chains compared to other bacteria [13, 14].

Annexins (ANXs) comprise a multigene superfamily and are known for their ability to bind to membranes in a calcium-dependent way. They are generally found in animal and plant kingdoms, and to date twelve ANX subfamilies are known in mammals, namely ANXA1-ANXA11 and ANXA13 [15, 16]. Extracellularly, ANXs interact with bacteria and viruses as well as with components of the extracellular matrix. They may play a regulatory role in inflammation, coagulation and fibrinolysis [17]. Some ANXs are able to interact with viruses and therefore may influence the infection process [17]. The interaction between ANXA5 and influenza virus appears to be essential for successful infection [18], similar to the interaction of ANXA2 with the human cytomegalovirus (CMV), human papilloma virus type 16 (HPV16) and enterovirus 71 (EV71) [19–21]. ANXA5 was also shown to bind some Gram-negative bacteria, such as *Pseudomonas aeruginosa*, *Shewanella putrefaciens* and *Haemophilus influenzae*, but not the previously tested Gram-positive bacteria, such as *Enterococcus faecalis*, *Streptococcus pyogenes* or *Streptococcus agalactiae*. Both *in vitro* and *in vivo* experiments displayed a possible role of ANXA5 in inhibiting the effect of LPS in endotoxin activities [22].

Although ANXs are present in the human stomach, little is known about their physiological function or their relevance for the *H*. *pylori* infection and pathogenicity. However, some associations between ANXs and gastric cancer have already been published [23–26]. Hence, this study was conducted to investigate an interaction of *H*. *pylori* and human ANXs, especially ANXA5. After developing a specific binding assay, we characterized the nature of binding and identified *H*. *pylori* lipid A as a bacterial binding target for ANXA5. Furthermore, we investigated whether the recognition by the host innate Toll-like receptor 4 (TLR4) is altered in bacteria covered by ANXA5. Possible effects of the ANX interaction regarding *cag*-T4SS function and translocation of CagA were also studied. Finally, human gastric tissue samples were analyzed to study the potential role of ANXs in complex infection situations and to gain a better understanding of the physiological significance of the binding.

## Results

### *H*. *pylori* binds ANXA5 and other members of the ANX family

**Fig 1:**
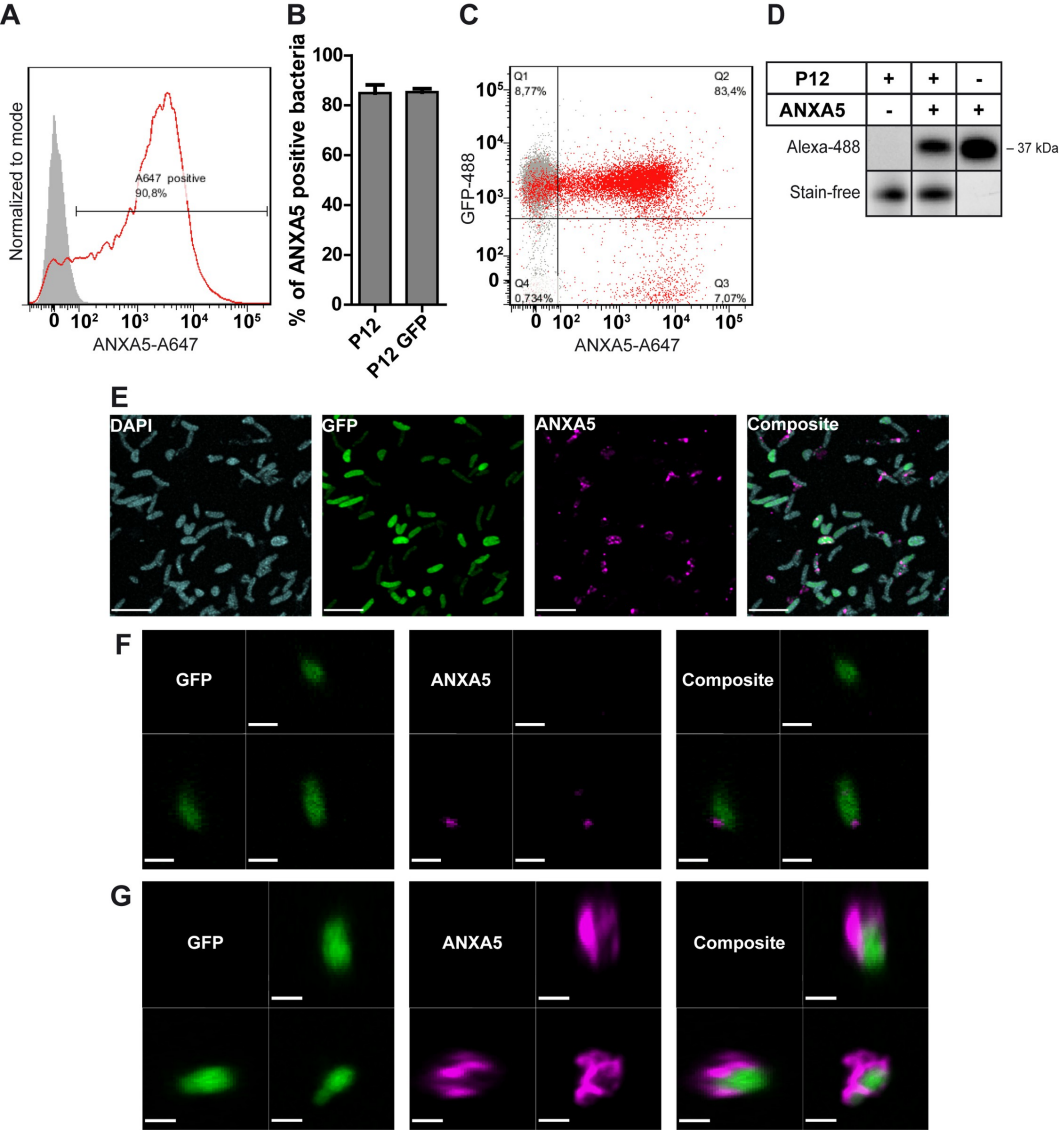
*H*. *pylori* is binding ANXA5. **A**) Representative flow cytometry histogram of the 647-fluorescence of *H*. *pylori* without (grey) and with ANXA5-Alexa 647 (A647) addition (red). **B**) ANX binding assay was performed with P12 or P12 GFP and ANXA5-A647. Flow cytometry data was gated to the ANXA5-positive events; the mean percentage of ANXA5-positive bacteria is shown with standard error of mean (SEM). Unpaired t-test was performed; n = 4–5. **C**) Dot plot showing ANXA5-A647- vs. GFP-488-channel of GFP-expressing *H*. *pylori* without (grey) and with (red) ANXA5-A647 addition. Quadrants are shown for the population with ANXA5-A647 addition (red). Q1 represents GFP-positive bacteria, Q2 represents GFP- and ANXA5 positive bacteria and Q3 represents ANXA5-positive bacteria. **D**) *H. pylori* and ANXA5-Alexa 488 (A488) were co-incubated and samples were loaded onto a polyacrylamide gel and analyzed under denaturing and reducing conditions. The ANXA5 band with a molecular weight of 37 kDa was detected by the Alexa 488 signal using the ChemiDoc MP system (BioRad). ANXA5-A488 was used as positive control. The stain-free gel is shown as a control for the amount of protein loaded. n = 3; one representative gel is shown. **E**) Analysis with the confocal SP8 microscope and STED technology; GFP-expressing *H*. *pylori* P12 strain (green) and ANXA5-Alexa 594 (magenta) were used; the fixed samples were stained with DAPI (blue). Scale bar 2.4 μm. **F, G**) Analysis with the confocal SP5 microscope and Volocity software; GFP-expressing *H*. *pylori* P12 strain and ANXA5-A647 were used. Scale bar equals 1 μm (F) and 1.3 μm (G).

To study a possible interaction of *H*. *pylori* with host cell ANXs, especially ANXA5, we first established and standardized an *in vitro* ANX binding assay. Therefore, GFP (green fluorescent protein)-expressing *H*. *pylori* strain P12 (P12-GFP) was incubated with fluorophore-coupled (Alexa-647) ANXA5 to determine and quantify ANX binding by flow cytometry. A strong increase of fluorescence indicated that *H*. *pylori* binds ANXA5 (Fig 1A) and quantification showed binding of ~85% of *H*. *pylori* P12 wild type (wt) and P12-GFP bacteria to fluorescent ANXA5 (Fig 1B).

To confirm that the events measured in the flow cytometer represented the intact bacteria binding ANXA5, P12-GFP was used to detect GFP-expressing bacteria together with Alexa-647 labelled ANXA5 (Fig 1C). The vast majority of bacteria showed a high 488 signal (Q1 and Q2), which indicates that P12 was expressing GFP, as expected. With ANXA5 addition, most events, which showed a positive signal in the 647-channel, were also positive in the 488-channel (83.4%), suggesting that intact bacteria bind the 647-labelled ANXA5. To further verify and strengthen the *H*. *pylori*-ANXA5 interaction by a biochemical method, *H*. *pylori* and ANXA5-Alexa 488 were co-incubated and samples were run by polyacrylamide gel electrophoresis. ANXA5 interaction was visualized by excitation and detection of its coupled fluorophore using a Gel Doc documentation system (ChemiDoc, BioRad) (Fig 1D). These data confirmed the flow cytometry data, supporting that *H*. *pylori* is binding ANXA5.

To gain information where on the bacterial surface ANXA5 localizes and how it is distributed, confocal laser scanning microscopy was performed. Since the GFP signal (green) was variable in intensity *H*. *pylori* P12-GFP was additionally stained with DAPI after fixation (Fig 1E). A high number of bacteria bound ANXA5 (magenta), correlating with the flow cytometry data. Whereas some bacteria bound only a small amount of ANXA5 in one spot (Fig 1F), others appeared completely “coated” with ANXA5 on their surface (Fig 1G).

**Fig 2:**
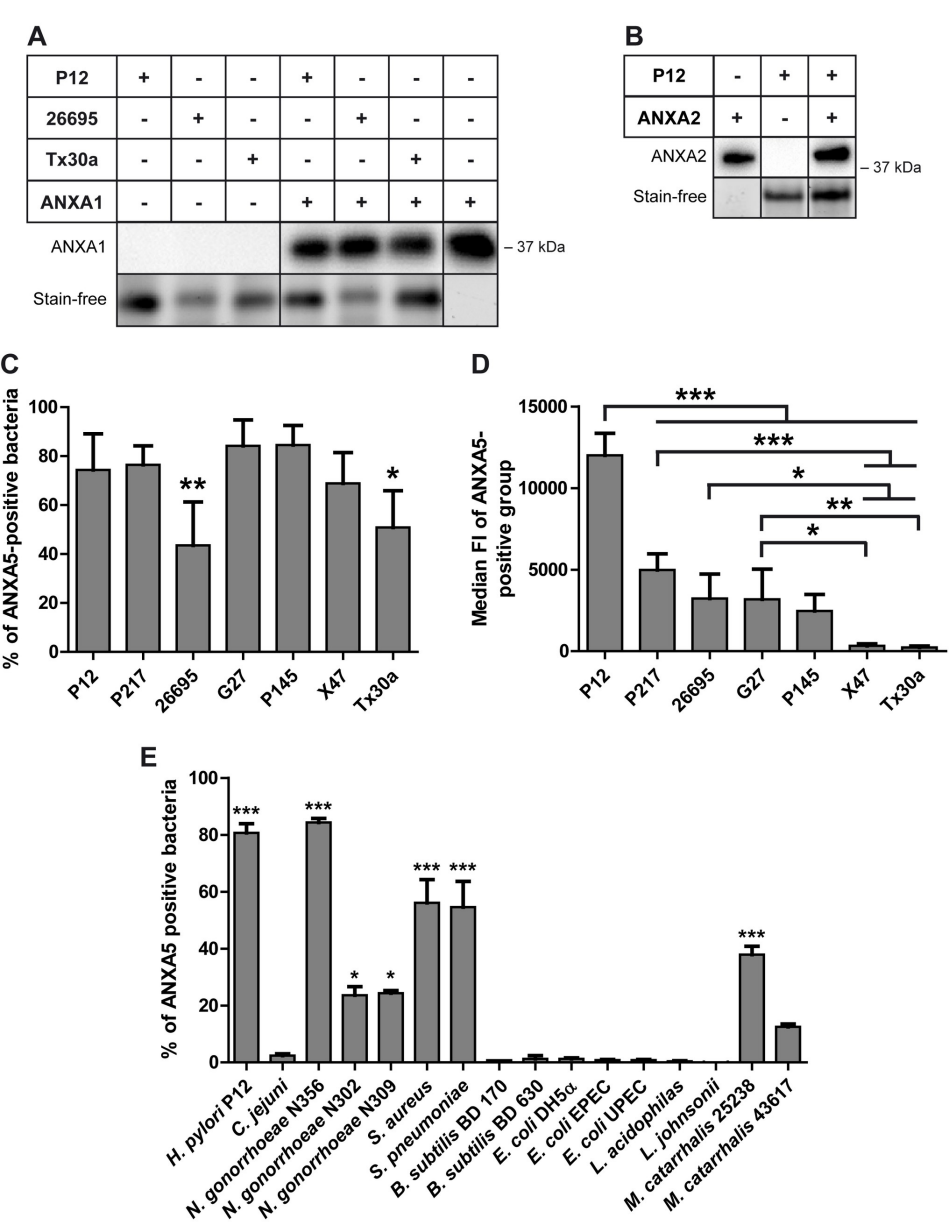
*H*. *pylori* binds ANXA1, ANXA2, and ANXA5, with ANXA5 binding being highly variable among different bacteria. **A**) *H*. *pylori* wt strains P12, 26695 and Tx30a were incubated with ANXA1, the samples were prepared, and gel electrophoresis under denaturing and reducing conditions and Western blots were performed using an anti-ANXA1 antibody (S3 Table) to detect the protein at 37 kDa. ANXA1 only was used as a positive control. The stain-free gel is shown as a control for the amount of protein loaded. n = 3; one representative gel is shown here. **B**) *H*. *pylori* P12 wt strain was incubated with ANXA2, the samples were prepared, and gel electrophoresis under denaturing and reducing conditions and Western blot were performed using an anti-ANXA2 antibody (S3 Table). ANXA2 only (37 kDa) was used as a positive control. The stain-free is shown as a control for the amount of protein loaded. One representative example is shown here. **C-E**) The ANX binding assay was performed using different *H*. *pylori* wt strains and ANXA5-A647 and analyzed by flow cytometry. **C**) Flow cytometry data was gated to the ANXA5-positive events; the mean percentage of ANXA5-positive bacteria is shown with standard deviation (SD). One-way ANOVA and Dunnett’s multiple comparison test against P12 as control were performed. n = 5 (n = 4 for 26695). * p<0.05; ** p<0.01; *** p<0.001. Representative histograms of the 647-fluorescence of the different bacteria without (grey) and with (red) ANXA5-A647 addition are shown in S1**D** Fig) The graphs show the mean value with SD of the median 647-fluorescence intensity of the gated “ANXA5-positive group”. One Way ANOVA, Post-hoc-test: Bonferroni’s Multiple Comparison Test, n = 5 (n = 4 for 26695). * p<0.05; ** p<0.01; *** p<0.001. **E**) ANX binding assay using different bacterial species and ANXA5-A647. The flow cytometry data were gated to the ANXA5-positive events; the mean percentage of bacteria in this group is shown here with SEM. *C*. *jejuni* is used as negative control for ANXA5 binding. All bacteria showing a significant (*) or highly significant (***) difference to the negative control are considered to bind ANXA5. A complete statistical analysis of ANXA5 binding of the different bacterial species shown shown in S2 Table. *** p<0.001, ** p<0.01. * p<0.05.

Next, we tested whether *H*. *pylori* would bind other members of the ANX family as well. ANXA1 and ANXA2 were selected, since they are described to play a role in gastric cancer and ANXA2 was found to be up-regulated in *H*. *pylori*-associated gastric cancer [23]. Fluorescently labelled ANXA1 or ANXA2 probes were not commercially available, therefore samples were run on a polyacrylamide gel and bound ANXs were detected by immunoblotting using corresponding antibodies. All *H*. *pylori* strains tested bound ANXA1 (Fig 2A) and ANXA2 (Fig 2B). In conclusion, by using different methods, such as flow cytometry, gel electrophoresis or confocal laser scanning microscopy, the data show that *H*. *pylori* is capable of binding human ANXs.

### High variability in ANX binding within *H*. *pylori* strains and other bacterial species

To study the nature of the *H*. *pylori*–ANX interaction in more detail, we now concentrated on ANAX5. Six additional *H*. *pylori* strains, type I (*cag*-PAI-positive) and type II (*cag*-PAI-negative) were analyzed with the ANX binding assay (for representative histograms see S1 Fig). All strains bound ANXA5, although the percentage of ANXA5-binding bacteria of a given strain was variable. While nearly ~80% of *H*. *pylori* P12, P217, P145, G27 and X47 bound ANXA5, only ~50% of 26695 and Tx30a bacteria showed binding (Fig 2C). We next determined the capacity of ANXA5 binding by the average bacterium using the median fluorescence intensity (FI) of the ANXA5-binding group of bacteria (see Experimental procedures for details). Surprisingly, this method of evaluation revealed that the P12 strain bound significantly higher amounts of ANXA5 than all other strains tested (Fig 2D) and that type II strains (X47 and TX30a) had the lowest binding. All together these data show that the ANXA5 binding is not unique for the P12 strain, but prevalent in all *H*. *pylori* strains tested. The ANXA5-binding capacity of individual bacteria varies considerably between independent isolates.

To clarify whether ANX binding is more common in bacteria, we included a set of 16 different (Gram-negative, Gram-positive, aerobic, microaerophilic, human pathogenic and nonpathogenic) bacteria in the binding assay (S1 Table for bacterial strains used, S2 Fig for histograms). Surprisingly, *C*. *jejuni*, a close relative to *H*. *pylori*, did not bind ANXA5 at all (Figs 2E and S2). Statistical analysis was performed comparing all bacteria with both, *H*. *pylori* P12 as positive and *C*. *jejuni* as negative control. Less than half of the tested bacterial species showed a significant difference in binding to *C*. *jejuni*, meaning that those bacteria did bind ANXA5 (Fig 2E and S2 Table). All strains except *Neisseria gonorrhoeae* N356 showed a significant difference in binding to *H*. *pylori* as the positive control, suggesting that they bound significantly less ANXA5 compared to *H*. *pylori* P12, or did not bind at all (Fig 2E and S2 Table).

### Growth conditions, but not cholesterol or serum affects the *H*. *pylori*–ANX binding

Next, we evaluated the effect of different growth conditions, especially liquid culture versus agar plate growth, which might influence the outer membrane lipid distribution or the “fitness” of the bacteria and could change the ANXA5-binding. The corresponding ANX binding assays showed a significant reduction in the ANXA5 binding capacity from ~50% of ANXA5-bound bacteria from plate-grown conditions to ~7% of bound bacteria grown in liquid culture. To exclude any interfering effect from the liquid culture medium on the binding, liquid culture medium was added to plate grown bacteria, which did not change the binding (S3A, S3C and S3D Fig). Taken together these experiments show that *H*. *pylori* most likely changes its surface binding characteristics depending on the growth conditions. These environmental changes apparently influence the binding of ANXA5.

*H*. *pylori* is known to extract cholesterol from its growth medium and integrates the glycosylated cholesterol into its membrane using the enzyme Cgt [27]. Furthermore, cholesterol can augment the binding of different ANXs, including ANXA5, to phospholipids [28]. To test whether cholesterol affects the binding of ANXA5, the ANX binding assay was performed with bacteria that had been cultivated on either serum, blood or cholesterol agar plates. Bacteria growing on cholesterol agar plates bound the most ANXA5, however, the quantitative analysis of the data showed no significant difference between bacteria grown on different plates (S3B and S3E–S3G Fig).

To further evaluate the role of cholesterol for the ANXA5 binding of *H*. *pylori*, a Δ*cgt* mutant was tested, which is not able to incorporate cholesterol into the bacterial membrane when grown on cholesterol-containing agar plates. We did not observe any difference in ANXA5 binding between the P12 wt and the mutant strain, neither in the amount of positive bacteria nor in the median FI. (S3H–S3K Fig). We therefore concluded that cholesterol incorporation into the bacterial membrane does not play an important role in the interaction of *H*. *pylori* and ANXA5.

### The *H*. *pylori*–ANXA5 interaction depends on calcium ions

Generally, ANXs can interact with eukaryotic membranes in at least three different ways: i) by calcium-dependent binding of their C-terminal domain to lipids, ii) by calcium-independent binding mediated by lipids and iii) directly via the N-terminal domain to soluble or membrane-associated proteins [15]. To determine the nature of the ANXA5 interaction with *H*. *pylori* these options were evaluated.

**Fig 3:**
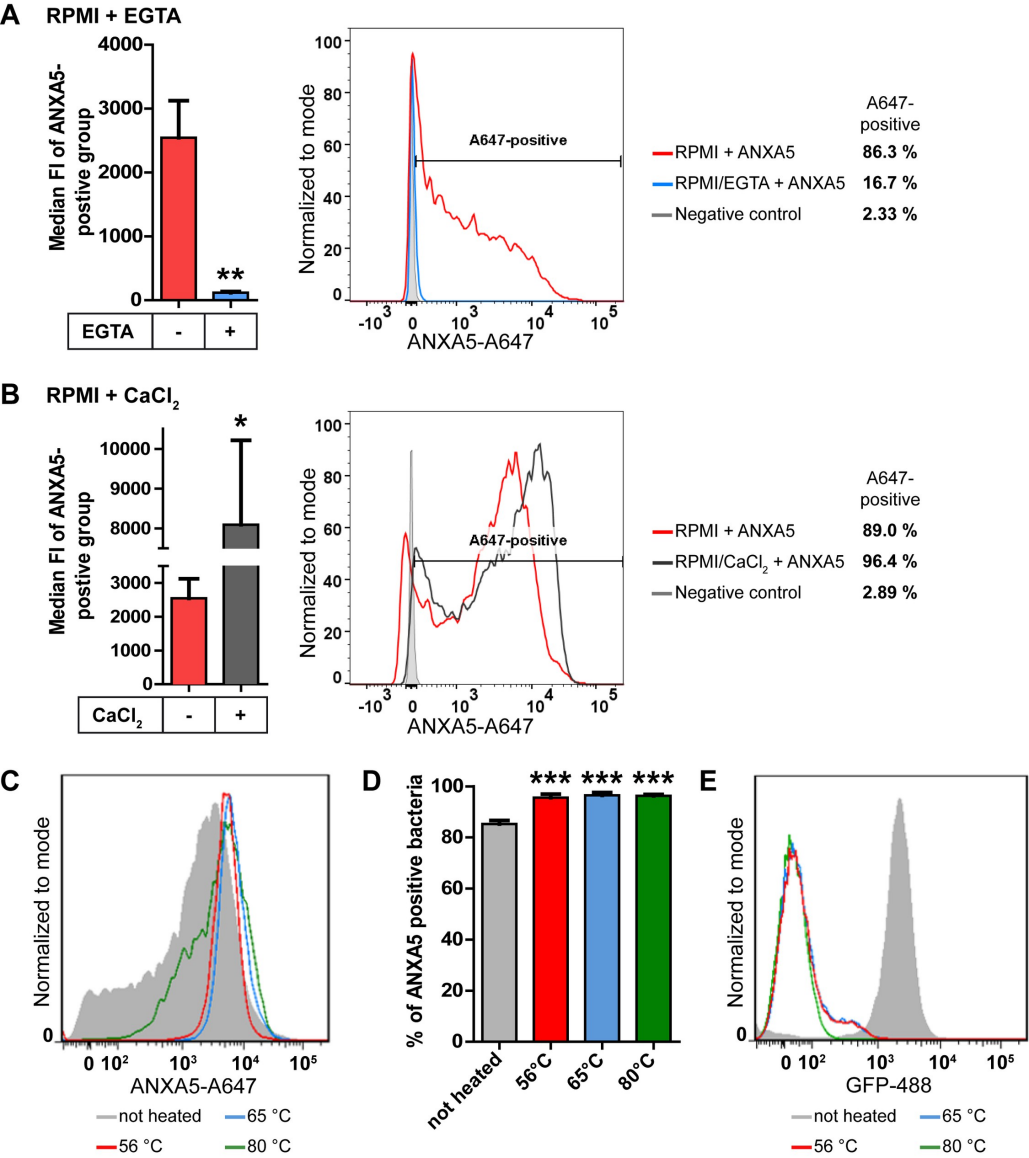
Effect of calcium, growth conditions and surface protein integrity on *H*. *pylori ANXA5* binding. **A**) The ANX binding assay was performed in RPMI with (blue) and without (red) 5 mM EGTA and ANXA5-A647. The flow cytometry data was gated to the ANXA5-positive events; the median FI of bacteria in this group is shown in the left graph ± SEM. Unpaired t-test was performed. n = 4. * p<0.05; ** p<0.01; *** p<0.001. One representative histogram of the 647-fluorescence of the bacteria with (blue) and without (red) EGTA is shown on the right. Unstained bacteria were used as a negative control (grey). **B**) ANX binding assay was performed with P12 in RPMI without (red) and with (dark grey) CaCl_2_ (final Ca^2+^-concentration: 0.42 and 1.8 mM, respectively) with ANXA5-A647. The flow cytometry data was gated to the ANXA5-positive events; the median FI of bacteria in this group is shown in the left graph ± SEM. Unpaired t-test was performed. n = 4. * p<0.05; ** p<0.01; *** p<0.001. A representative histogram of the 647-fluorescence of the bacteria with (dark grey) and without (red) CaCl_2_ is shown on the right. Unstained bacteria were used as a negative control (light grey). **C-E**) ANX binding assay was performed with untreated bacteria (grey) or with bacteria heated to 56°C (red), 65°C (blue) or 80°C (green) with ANXA5-A647. **C**) A representative histogram of the 647-fluorescence of the heated bacteria. **D**) Flow cytometry data were gated to the ANXA5-positive events; the mean percentage of bacteria in this group are displayed here ± SEM. Non-heated bacteria (grey) were compared to bacteria with protein denaturation. Statistical analysis with one-way ANOVA and Dunnett’s multiple comparison post-test against the non-treated control; n = 3–4. * p<0.05; ** p<0.01; *** p<0.001. **E**) Representative histogram of the 488-fluorescence of the bacteria. This was used as a control for protein denaturation, as the GFP protein was destroyed by heating. Same color code used as in (C/D).

First, the role of calcium ions was tested, since the commonly known way of interaction between ANXs and membrane phospholipids is calcium-dependent [29]. Hence, the ANX binding assay was performed with the chelator EGTA, which binds divalent ions and shows a high affinity for calcium ions. When EGTA was added, the median FI decreased drastically, indicating that *H*. *pylori* bound hardly any ANXA5 (Fig 3A).

Additionally, we tested whether an increased calcium concentration would have any effect on the ANXA5 binding. Therefore, CaCl_2_ was added to RPMI, so that RPMI had the same calcium concentration as the standard ANX binding buffer (1.8 mM) specifically designed to investigate ANX-interactions [30]. Under these conditions higher amounts of ANXA5 were bound on average, as indicated by the increase of the median FI (Fig 3B). In conclusion, when more calcium is present, bacteria bind higher amounts of ANXA5. These findings strongly indicate that the *H*. *pylori*—ANXA5-interaction is calcium-dependent and the concentration of calcium present plays an essential role.

### Further binding studies suggest ANXA5 binding to lipopolysaccharide of *H*. *pylori*

To discriminate between a protein and a lipid as binding target of ANXA5 on the surface of *H*. *pylori*, the bacteria were exposed to protein denaturation by heating to different temperatures. Heated bacteria displayed a slightly higher ANXA5 binding (Fig 3C) and a significantly higher number of ANX5-positive bacteria (Fig 3D). The successful heat denaturation of proteins was controlled by using the P12-GFP strain showing a positive GFP signal, whereas the temperature-treated bacteria did not show any GFP signal (see representative histogram, Fig 3E). Taken together these data suggest that the ANXA5 binding of *H*. *pylori* is not dependent on the integrity of bacterial proteins and is therefore most likely lipid mediated. The increased binding of ANXA5 to protein-denatured bacteria might be explained by its better access to membrane lipids.

**Fig 4:**
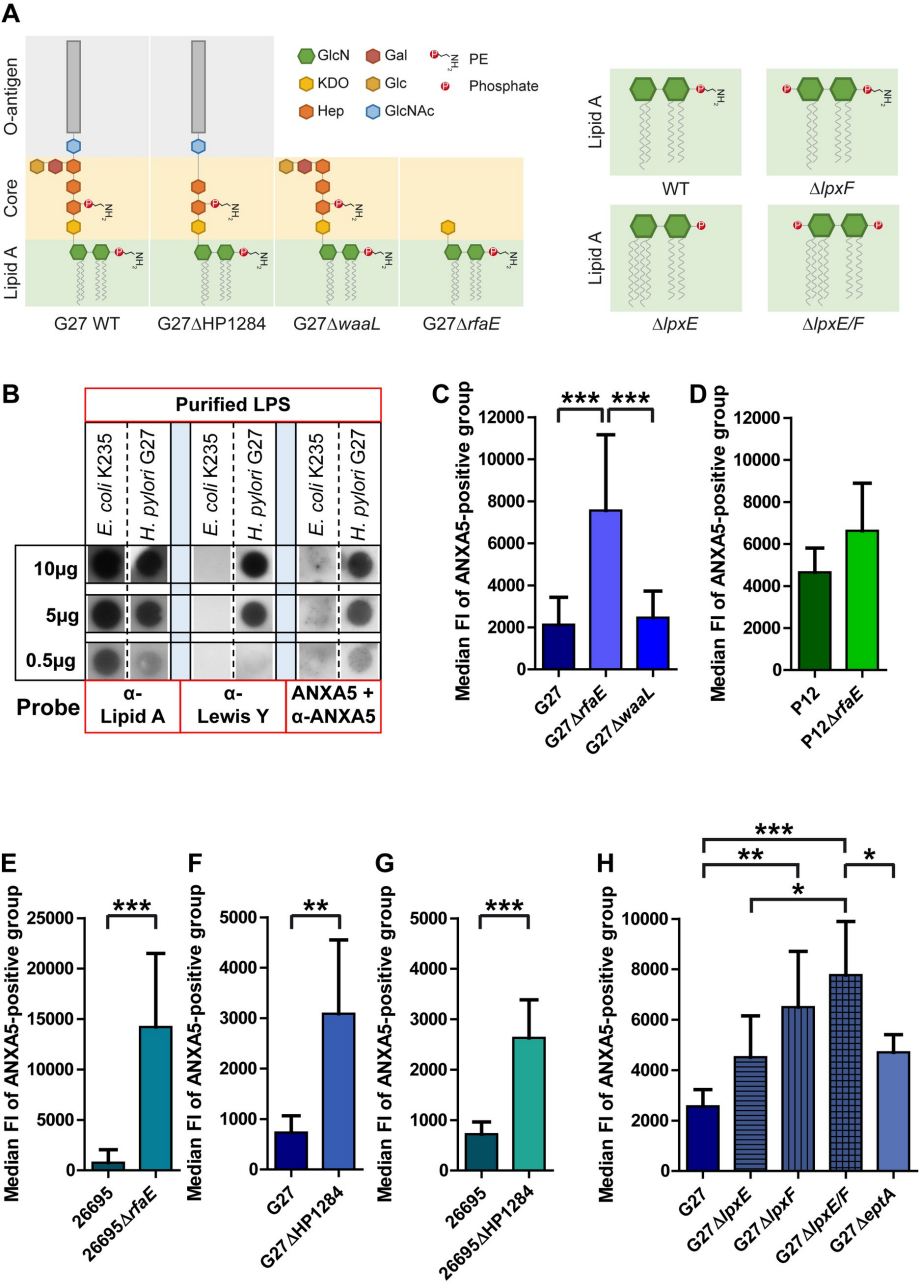
Characterization of lipid A as binding target for ANXA5. **A**) Overview of LPS structures of G27 wt, G27ΔHP1284, G27Δ*waaL* and G27Δ*rfaE* (left, adapted from [31]) and lipid A phosphorylation status of *H*. *pylori lpx* mutant strains (right, adapted from [10]). GlcN–glucosamine; Gal–galactose; KDO– 3-deoxy-D-manno-oct-2-ulosonic acid; Glc–glucose; Hep–heptose; GlcNAc–*N*-acetylglucosamine. **B)** The binding of ANXA5 to LPS from *E*. *coli* and *H*. *pylori* was analyzed by dot blot. Different amounts of LPS (10, 5, and 0.5 μg) on a PVDF membrane were incubated with anti-lipid A antibody, anti-Lewis Y, or ANXA5 (2 μg/ml, A9460, Sigma) followed by anti-ANXA5 antibody (for antibodies used see S3 Table). Lipid A and Lewis Y were used as loading controls. Shown is a representative image from at least 3 independent experiments. **C-G**) Different *H*. *pylori* wt and LPS mutant strains were analyzed for ANXA5-Alexa647 binding to G27 and respective mutants (C, F), P12 and respective mutants (D), and 26695 and respective mutants (E, G). Results are depicted as mean ± SD. Statistics were calculated using one-way ANOVA with Bonferroni’s multiple comparison test (C) or Student’s unpaired t-test (D-G). n = 8 (C), n = 5 (D), n = 7± (E), n = 6 (F), n = 5 (G). * p < 0.05, ** p < 0.01, *** p < 0.001. Representative histograms are shown in Fig S5. **H**) Median FI of ANXA5-positive group of G27 wt, Δ*lpxE*, Δ*lpxF*, Δ*lpxE/F* and Δ*eptA* strains was analyzed as described before. Results are depicted as mean ± SD. Statistics were calculated using one-way ANOVA with Bonferroni’s multiple comparison test. n = 6 (n = 7 for Δ*lpxE*; n = 5 for Δ*lpxF*). * p < 0.05, ** p < 0.01, *** p < 0.001.

Binding of ANXA5 to LPS of a few Gram-negative bacteria was described previously [22]. *H*. *pylori* as a Gram-negative bacterium contains LPS on its outer membrane, although its structure is unique [31]. We used highly purified LPS from *H*. *pylori* strain G27, the structure of which has been determined recently [32] (Fig 4A). In a dot blot assay, the binding of ANXA5 was compared to LPS of *E*. *coli* K-235 as a negative control (Fig 4B). An anti-Lipid A antibody verified the binding of LPS on the membrane (0.5 μg, 5 μg or 10 μg), whereas the anti-Lewis Y antibody specifically recognized the corresponding epitope present on *H*. *pylori* G27, but not on *E*. *coli* K-235 LPS (Fig 4B), which is devoid of this epitope. Incubation with ANXA5 and detection with an anti-ANXA5 antibody revealed binding to *H*. *pylori*, but not *E*. *coli* LPS, demonstrating a specific, concentration-dependent interaction of ANXA5 with *H*. *pylori* LPS (Fig 4B).

### The *H*. *pylori* lipid A/core region is a ligand for ANX binding

To further localize and narrow down the ANXA5 binding target on *H*. *pylori* LPS, we applied a set of *H*. *pylori* strains with inactivation of defined genes encoding key proteins in the LPS biosynthesis pathway (see Fig 4A for a schematic overview of mutant LPS structures). First, the LPS mutant strains G27Δ*waaL* and G27Δ*rfaE* were used, which either lack the complete LPS O-antigen (Δ*waaL*) or are deficient in the heptose sugars (Δ*rfaE*), connecting the O-antigen and core structure to the lipid A membrane anchor, respectively (Figs 4A and S4A).

The G27Δ*waaL* and *rfaE* mutant strains bound only slightly more ANAX5 (97.1% and 99.8%) as compared to the G27 wt strain (94.9%) (S5A Fig). However, the median FI of the events from the ANXA5-positive group was significantly higher in the G27Δ*rfaE* versus the G27 wt or G27Δ*waaL* mutant strain (Fig 4C), indicating that ANXA5 can bind *H*. *pylori* G27 significantly better when the core structure and the complete O-antigen of LPS are absent. To verify these results with other *H*. *pylori* strains, we used *H*. *pylori* P12, 26695 and the corresponding Δ*rfaE* mutants (S4C Fig). For P12Δ*rfaE* the ANXA5 binding was higher as compared to the P12 wt strain (90.5% versus 72.9%) (S5B Fig). The median FI of the events from the ANXA5-positive group was slightly higher in the P12Δ*rfaE*, but not significantly different to the P12 wt strain (Fig 4D). A striking difference in ANXA5 binding was seen between the 26695 wt (22.0%) and the 26695Δ*rfaE* mutant strain (85.9%) (S5C Fig), which also was supported by a significant difference between the median FI of the ANXA5-positive group of both strains (Fig 4E).

These results could be corroborated further by using *H*. *pylori* strains harboring a deletion in the heptose III transferase gene (ΔHP1284) in strains G27 and 26695. These mutations result in an incomplete core region, like the *rfaE* mutants (Figs 4A and S4A and S4C). ANXA5 binding revealed a significantly higher median FI from the ANXA5-positive group in the ΔHP1284 versus the corresponding wt strains (Figs 4F–4G and S5D–S5E), indicating that ANXA5 can bind *H*. *pylori* G27 and 26695 significantly better when part of the core structure, especially the third heptose and the adjoining Glc-Gal residues are missing, although the O-antigen of LPS is still present (Fig 4A) [31]. In conclusion, the corresponding ΔHP1284 mutants behaved like the *rfaE* mutants concerning ANAX5 binding, whereas the *waaL* mutant, which harbors a complete core structure, but lacks the complete O-antigen, behaves like the wt strain concerning ANXA5 binding. These data suggest that the O-antigen of LPS does not significantly influence ANXA5 binding, but changes in the core region (ΔHP1284 and *rfaE* deletions) seem to expose the binding target of ANXA5 on LPS.

### The lipid A phosphorylation status determines ANXA5 binding of *H*. *pylori*

Since the ANXA5 interaction with *H*. *pylori* lipid A is strongly calcium-dependent, we speculated that negatively charged structures on lipid A might be crucial for ANXA5 binding. We therefore concentrated on the phosphate groups of lipid A. In the process from lipid A synthesis to outer membrane insertion an originally bisphosphorylated form of lipid A is modified to result in a mature major lipid A species lacking both, the 4´-phosphate (removed by LpxF) and the 1-phosphate group (cleaved by LpxE), the latter being subsequently substituted with a phosphoethanolamine residue catalyzed by EptA [10]. We generated *lpxE* and *lpxF* mutants as well as *lpxE/F* double mutants in *H*. *pylori* G27 (see Fig 4A for a structural overview on *lpx* mutant strains), since this strain is best characterized concerning its LPS structure [33]. In addition to *lpxE* and *lpxF*, we also inactivated the *eptA* gene encoding the enzyme which transfers phosphoethanolamine to the *H*. *pylori* lipid A. The intact LPS structure of these mutants was verified by SDS PAGE and silver staining (S4B Fig).

ANXA5 binding studies revealed a significant increase in ANXA5 binding for both, the G27Δ*lpxF* mutant and a G27Δ*lpxE/F* double mutant as compared to the G27 wt strain (Fig 4H). A G27 *lpxE* deletion strain showed a stronger tendency for ANXA5 binding without reaching statistical significance. The absence of the *eptA* gene in G27 resulted in an ANXA5 binding pattern similar to the *lpxE* mutant.

In conclusion, the binding assays suggest that ANXA5 binding of strain G27 is significantly enhanced when its lipid A is phosphorylated at the 4´-sugar position. However, the 1-phosphate, as well as the lack of a phosphoethanolamine at this position, both seem to slightly support ANXA5 binding.

### ANXA5 binding inhibits LPS-mediated TLR4 signaling

In response to bacterial infection, the infected host produces antimicrobial proteins, such as lactoferrin, lipocalin or calprotectin (CP), which sequester soluble metal ions [34, 35]. Especially CP can sequester the transition metals iron, manganese and zinc via chelation. To promote resistance to CP *H*. *pylori* alters its lipid A molecules and changes its growth behavior, which also results in increased biofilm formation [36]. Interestingly, *H*. *pylori* evades recognition of the host’s immune system by modifying lipid A, especially by removing the phosphate groups, as described above [10]. Structural and experimental infection studies indicate that especially the 4´-phosphate group is important for TLR4-MD2 recognition of lipid A [10, 37]. We therefore hypothesized that *H*. *pylori* might use ANXs binding to shield its phosphorylated lipid A molecules against the recognition by the TLR4-MD2 complex from cells of the innate immune system or epithelial cells in the stomach mucosa.

**Fig 5:**
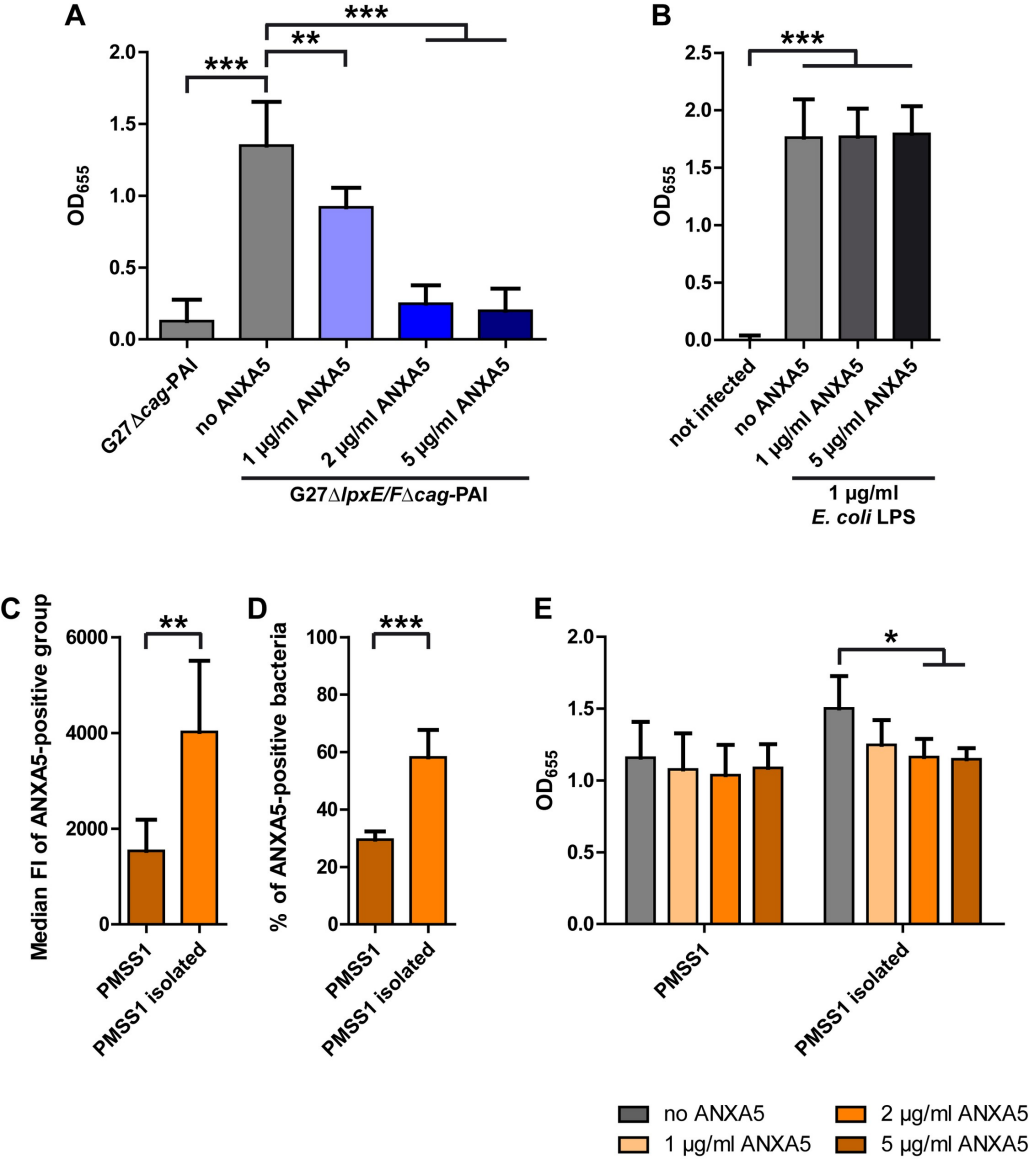
Influence of ANXA5 binding on TLR-4 activation by different *H*. *pylori* strains. **A, B, E**) HEK-Blue-hTLR-4 cells were infected with (A) G27Δ*cagPAI* or G27Δ*lpxE/F*Δ*cagPAI*, or with (E) PMSS1 or re-isolated PMSS1. **B**) *E*. *coli* K-235 LPS was used as positive control and uninfected cells served as negative control. NF-κB signaling was determined using QuantiBlue (Invivogen) and OD was measured at 665 nm. Results are depicted as mean ± SD. **C-D**) ANXA5-binding was analyzed for *H*. *pylori* PMSS1 or a mouse-isolated PMSS1 strain by flow cytometry. Data were evaluated for median FI (C) and percentage of ANXA5-positive bacteria (D). Results are depicted as mean ± SD (A). Statistics (A-B) were calculated using one-way ANOVA with Bonferroni’s multiple comparison test or two-way ANOVA (E) with Bonferroni’s post hoc test. Statistics (C-D) were analyzed using Student’s t-test. n = 5 (A, B-D), n = 4 (E). * p < 0.05, ** p < 0.01, *** p < 0.001.

To study the role of ANX binding to *H*. *pylori* lipid A for its recognition by the TLR4-MD2 complex we monitored TLR4 activation using HEK293 cells stably transfected with the TLR4 machinery (HEK-Blue-hTLR4) and a secreted alkaline phosphatase reporter gene controlled by an NF-kB inducible promoter (Invivogen). This colorimetric assay allows to quantify TLR4-MD2 complex activation. To eliminate the contribution of the *cag*-PAI on activation of the NF-kB promoter, we generated a Δ*lpxE/F* double mutant with the subsequent deletion of the entire *cag*-PAI (G27Δ*lpxE/F*Δ*cag*-PAI). Co-incubation of *H*. *pylori* G27Δ*lpxE/F*Δ*cag*-PAI with the reporter cell line clearly showed a significantly higher activation of the NF-kB promoter as compared to the G27Δ*cag*-PAI strain. Remarkably, a significant, concentration-dependent reduction in activation of hTLR4-MD2 in G27Δ*lpxE/F*Δ*cag*-PAI was observed upon addition of ANXA5 from 1 to 5 μg/ml (Fig 5A).

To determine the specificity of the hTLR4-MD2 activation by *H*. *pylori* we incubated the reporter cell line with purified *E*. *coli* LPS (1 μg/ml), which clearly stimulated the reporter cell activity. In contrast, addition of ANXA5 (1 or 5 μg/ml), which does not bind *E*. *coli* LPS (Fig 4B), did not have any effect on the NF-kB promoter activity, ruling out any direct inhibitory activity of ANXA5 on the hTLR4-MD2 complex (Fig 5B).

### Gastric mouse colonization significantly enhances the *H*. *pylori* ANXA5 binding capacity to interfere with TLR4 signaling

To test whether *H*. *pylori* grown under *in vitro* conditions on agar plates would change its surface in favor of ANXs binding during a stomach passage we infected mice with the mouse adapted *H*. *pylori* strain PMSS1 for three weeks and tested the ANXA5 binding before infection and immediately after re-isolation. Notably, the ANXA5 binding capacity was twice as high in the re-isolates compared to the pre-infection strain. This was the case for both the median FI of the ANXA5-positive group (Fig 5C) and the total percentage of ANXA5-binding bacteria (Fig 5D). The ability of PMSS1 re-isolates to stimulate TLR4 was increased compared to the pre-infection strain (Fig 5E), but the difference was not significant, presumably because the *cag*-T4SS of this wt strain also strongly contributes to the stimulation of NF-kB. Nevertheless, TLR4-MD2 specific stimulation in the reporter cell line could be significantly inhibited with ANXA5 in the re-isolates, but not in the pre-infection strain (Fig 5E). Taken together these data suggest that the ability of *H*. *pylori* to bind ANXs can be upregulated in the mouse model *in vivo*, presumably by changes in the lipid A phosphorylation status.

### *H*. *pylori*-ANXA5 interaction reduces CagA translocation into AGS cells but does not affect cell binding or IL-8 induction

**Fig 6:**
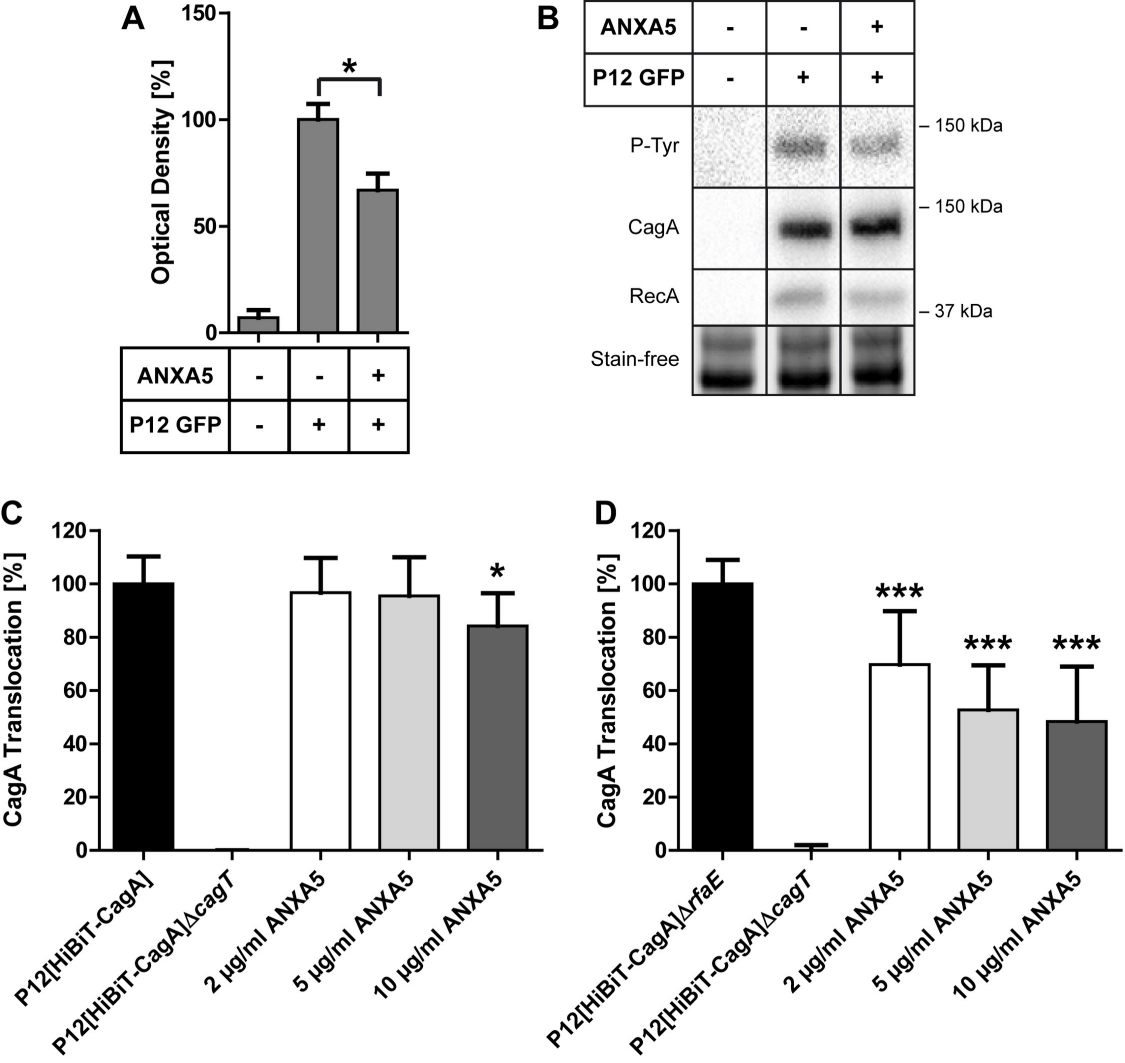
Effect of ANXA5 binding on CagA translocation of *H*. *pylori*. **A-B**) AGS cells were infected with *H*. *pylori* strain P12-GFP. *H*. *pylori* has been pre-incubated with ANXA5 before infection. **A**) Shown is the optical density of the CagA P12-GFP P-Tyr band with ANXA5 pre-incubation. Quantification of CagA P-Tyr was performed using ImageLab software (Version 6.0.1, BioRad) and normalized to the stain-free gel as shown in (B). **B**) Representative blots of phosphorylated CagA (P-Tyr), CagA and RecA as well as the stain-free-image are shown. Statistical analysis was performed with one-way ANOVA and Bonferroni’s post test; n = 4. * p<0.05; ** p<0.01; *** p<0.001, **C-D**) CagA translocation was measured using the HiBiT translocation assay with P12[HiBiT-CagA] (C) and P12[HiBiT-CagA]Δ*rfaE* (D). P12[HiBiT-CagA]Δ*cagT* was used as negative control. 1 h prior to infection, 2, 5 or 10 μg/ml ANXA5 were added to the bacteria. Results are shown as mean ± SD. Statistics were calculated using one-way ANOVA with Dunnett’s multiple comparison test. n = 5. (D), n = 5 (E, n = 6 for 2 μg/ml, 5 μg/ml). * p < 0.05, ** p < 0.01, *** p < 0.001.

Earlier experiments reported that the addition of ANXA5 to an infection of *H*. *pylori* with the gastric epithelial cell line AGS resulted in a ~20% decrease of CagA translocation [38]. When *H*. *pylori* was pre-incubated with ANXA5 and was then used to infect AGS cells, CagA translocation was significantly reduced (~33% on average) (Fig 6A and 6B). To verify these results, we applied phosphorylation–independent, quantitative CagA translocation assays. The TEM1-beta-lactamase reporter assay [39] was not applicable, due to the necessity of high calcium concentrations needed for ANXA5 binding, which interfered with the reporter assay. Therefore, we next applied the HiBiT-CagA translocation reporter assay developed and adapted for *H*. *pylori* recently [40]. This luciferase-based assay, making use of an N-terminal fusion of CagA with an 11-residue split-luciferase (HiBiT) tag, demonstrated a significant reduction of CagA translocation into AGS cells (~20%) in an ANXA5 concentration-dependent manner (Fig 6C). We next used a P12[HiBiT-CagA]Δ*rfaE* mutant strain for determination of CagA translocation into AGS cells. Notably, this mutation, which results in a major disturbance of the *H*. *pylori* cell envelope, apparently also affected the integrity of the *cag*-T4SS, resulting in a lower CagA translocation efficiency compared to the wt strain. However, an even stronger effect of roughly 50% reduction in CagA translocation was seen in comparison of the P12 wt versus the P12[HiBiT-CagA]Δ*rfaE* mutant loaded with 10 μg/ml ANXA5 (Fig 6D), suggesting that the unmasking of lipid A and the higher amount of ANXs binding directly affects CagA translocation efficiency of *H*. *pylori*.

We next studied the possible effects of *H*. *pylori* ANXA5 binding to the bacteria-host cell interaction. Notably, the binding of the bacteria to AGS cells was not at all affected when ANXA5 was added simultaneously or by pre-incubation of the bacteria (S6A and S6B Fig). Moreover, ANXA5 addition had no effect on IL-8 production of AGS cells by the *cag*-T4SS (S6C and S6D Fig). In summary, our data indicate that ANXA5 binding to *H*. *pylori* LPS can reduce the CagA translocation efficiency without affecting the bacterial binding capacity to host cells.

### *H*. *pylori* infected gastric tissue reveals elevated levels of ANXA2/5 and its binding/recruitment to the bacterial surface

**Fig 7:**
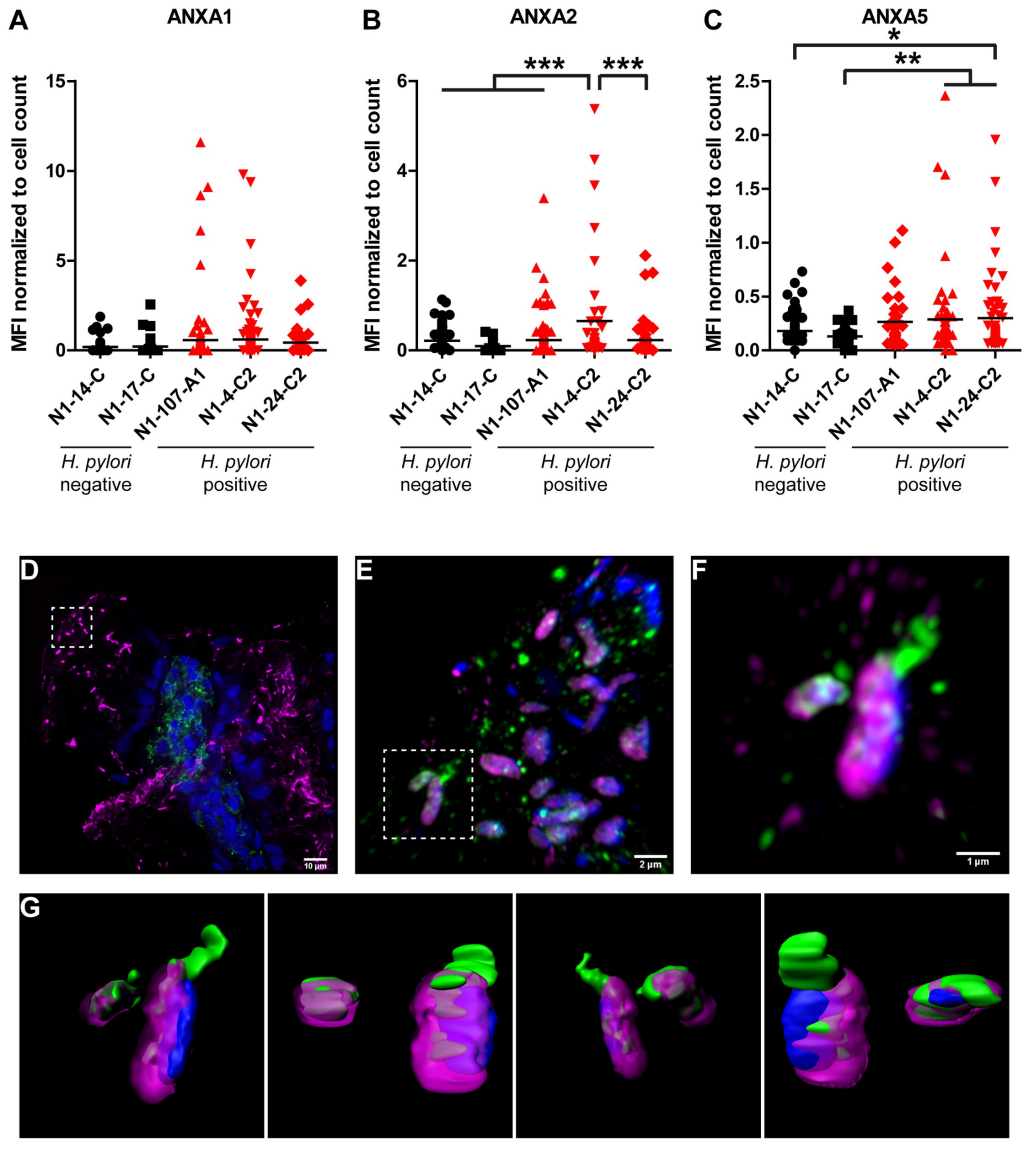
Elevated levels of ANXs in *H*. *pylori* infected versus non-infected human stomach biopsy sections and co-localization *in vivo*. **A-C**) ANXA1 (A), ANXA2 (B) and ANXA5 (C) levels were quantified in *H*. *pylori* positive (red) and negative (black) gastric biopsy samples. Gastric sections from antrum (N1-107-A1) or from corpus (N1-14-C, N1-17-C, N1-4-C2, N1-24-C2) were stained with antibodies as described in Material and Methods (*Immunohistochemistry of tissue slides…)*. Sections were imaged by the Leica TCS SP5 confocal microscope and quantification was performed using Fiji software. Data analysis was as described in Materials and Methods. Statistical analysis was performed using one-way ANOVA with a Bonferroni’s Multiple Comparison test. * p < 0.05, ** p < 0.01, *** p < 0.001. **D-G**) Images were acquired by super resolution confocal microscope LSM 880 (Zeiss) with Airyscan module. (D) Overview imaging showing *H*. *pylori* (magenta), ANXA5 (green) and bacteria (blue). The *H*. *pylori* analyzed further is indicated by white doted square. (E) Confocal image showing *H*. *pylori* (magenta/blue), co-localized with ANXA5 (green) and bacteria (blue). (F) High magnification image from cropped indicated area in (E), showing the localization of ANXA5 on the *H*. *pylori* surface. (G) 3D rendering images from image (F) showing the imaged in diverse rotated angles, for the distribution of annexin on the membrane surface of *H*. *pylori*. Scale bars are displayed. Imaris software was used.

To investigate whether the *H*. *pylori* infection in humans might affect production and release of ANXs in the gastric mucosa, gastric sections were stained with primary antibodies against ANXs A1, A2 and A5 and the amount of ANXs present was determined by the fluorescence intensity of the anti-ANXA 1, 2 and 5 antibodies calculated per cell (see Experimental procedures for details). Analysis of gastric antrum and corpus tissue sections from two non-infected and three infected individuals showed a slightly enhanced amount of ANXA1 in *H*. *pylori*-positive versus *H*. *pylori*-negative tissue, which did not reach statistical significance (Fig 7A). For ANXA2 and ANXA5 a significantly higher quantity of green fluorescence per cell nucleus was detected, indicating a higher amount of ANXA2 and ANXA5 in gastric corpus, but not gastric antrum tissue of infected versus non-infected individuals (Fig 7B and 7C).

To test whether *H*. *pylori* would also bind ANXs under *in vivo* conditions in the gastric mucosa, human *H*. *pylori* infected gastric corpus sections were stained with anti-*H*. *pylori* antiserum (magenta), anti-ANXA5 antibody (green), and DAPI to visualize host cell tissue. As controls, sections were stained for *H*. *pylori* and the secondary antibodies of the ANXA5 staining (goat anti-mouse IgG-Alexa488) or with both secondary antibodies only (S7A and S7B Fig). Indeed, co-localization of *H*. *pylori* and ANXA5 was detected in *H*. *pylori-*positive patient sections using confocal laser scanning microscopy (Fig 7D–7F). The green signal (ANXA5) overlaps with the magenta signal (*H*. *pylori*), demonstrating an interaction of the bacteria with ANXA5 *in vivo*. High resolution confocal images of two individual bacteria were taken with z-stacks and 3D projections rendered images. Displayed images with different rotated angles (y-axis) show cell volume with close binding of ANXA5 mostly covering the bacterial pole (Fig 7G and S1 Movie).

In conclusion, these data suggest that the *H*. *pylori* infection results in a higher abundance of ANXs in the gastric tissue and the colonizing *H*. *pylori* are able to recruit these ANXs to their surface.

## Discussion

ANXs are soluble proteins and are mainly found in the cytosol of eukaryotic cells. However, some ANXs such as A1, A2, A4 and A5 can also be detected extracellularly. They are primarily known to interact with eukaryotic membranes and modulate membrane trafficking and organization, including exo-, endo-, and phagocytic processes [15]. Recombinant ANXA5 is commonly used to detect the presentation of phosphatidylserine (PS) on the outer leaflet of the cell membrane, since externalized PS serves as a recognition and clearance signal for phagocytes to identify apoptotic cells [41].

We describe here for the first time the intense interaction between ANXs and *H*. *pylori*. After having initially established the binding of *H*. *pylori* to ANXA5, we standardized the assay and investigated the nature of this interaction using different methods. In addition to ANXA5, ANXA1 and ANXA2 were also shown to be bound by *H*. *pylori*. To demonstrate a more general relevance of our results, we extended the binding studies to a number of other *H*. *pylori* strains as well as some other Gram-negative and Gram-positive bacterial species. Interestingly, some species showed significant binding, while others showed no binding at all (Fig 2E and S2 Table). We then identified the lipid A portion of *H*. *pylori* LPS as a binding target for human ANXA5. Earlier work already showed that LPS from *cag*-PAI positive type I *H*. *pylori* strains induced a stronger TLR4 signaling in cognate target cells as compared to LPS of type II strains [42]. Intrinsic differences in the structure or bioactivity of LPS dependent on the presence of the *cag*-PAI might explain the generally lower ANXA5 binding of type II strains in our assays.

At this point, it is unclear why *E*. *coli* and *C*. *jejuni* did not bind ANXA5, even though they carry a bis-phosphorylated lipid A disaccharide, which in *C*. *jejuni* is masked by phosphoethanolamine that might interfere with ANXs binding. The fact that Gram-positive *S*. *aureus* and *S*. *pneumoniae* clearly showed binding of ANXA5 argue against a general or exclusive binding of ANXs to lipid A. Possible other targets might be lipoteichoic acid in case of Gram-positive bacteria, or membrane components, such as PS or other membrane lipids. Thus, also for *H*. *pylori*, lipid A might not be the only possible target for ANX interaction, since P12 did not significantly change its binding pattern upon deletion of *rfaE* (Fig 4D), suggesting that ANXA5 might have an additional binding target in this strain besides lipid A.

To evade the host, innate immune system some pathogenic bacteria, including *H*. *pylori*, apparently change their surface characteristics and therefore employ different strategies. During LPS biogenesis in *H*. *pylori* and some other bacteria, the negatively charged phosphate groups on the lipid A disaccharide backbone (1-phosphate) are subsequently removed and replaced by the more neutral substituent phosphoethanolamine [10, 43]. As a result, negative surface charges are lost, leading to a bacterial membrane that is more resistant to cationic antimicrobial peptides and proteins [44].

We show that the conserved LPS core region, but not the O-antigen chain, interferes with ANXA5 to access its lipid A binding target by using a series of defined *H*. *pylori* mutant strains in key genes of the LPS biosynthetic pathway. Due to the strict calcium dependence of ANXA5 binding to *H*. *pylori*, a negative-charged structure, such as the phosphorylated lipid A sugars, were considered as possible target, and therefore *lpxE/F* mutants were generated and tested. As expected, the 4´-phosphate, but also the 1-phosphate group of lipid A play an important role for ANX binding. The gene deletion–mediated prevention of *H*. *pylori* lipid A dephosphorylation by phosphatases LpxE, LpxF or both together, which results in mono- or bis-phosphorylated lipid A, respectively [10], significantly increased the binding of ANXA5 in strain G27 (Fig 4H). Furthermore, the presence of phosphorylated lipid A clearly enhanced the recognition of *H*. *pylori* LPS by the innate immune receptor TLR4, as already shown by Cullen *et al*. [10]. In addition to *lpxE* and *lpxF*, we also inactivated the *eptA* gene, which encodes the enzyme that transfers a phosphoethanolamine residue to the 1-phosphate after its dephosphorylation by LpxE. This resulted in a similar slightly enhanced ANXA5 binding comparable to the G27Δ*lpxE* mutant (Fig 4H), suggesting that the negatively charged phosphate groups, rather than the neutral phosphoethanolamine residue, plays an important role in strain G27 ANX binding.

This strategy of *H*. *pylori* to evade innate immunity by reducing the surface charge of lipid A seems to be generally straight forward. However, in the human stomach *H*. *pylori* comes in contact with activated neutrophils, which release large amounts of antimicrobial proteins, such as CP [36], comprising as much as 60% of the soluble protein content of the cytosol of a neutrophil [45]. CP sequesters soluble metal ions at the pathogen-host interface, designated as nutritional immunity [35]. This host response causes *H*. *pylori* to alter its lipid A molecules, apparently by a posttranscriptional mechanism, including the inactivation of LpxF, which results in an increased phosphate decoration of the outer membrane. This programmed surface change of *H*. *pylori* in the presence of CP was reported to induce a better bacterial resistance and fitness against the antimicrobial protein and induces a strong biofilm formation [36].

Thus, under infection conditions and in contact with CP released by activated neutrophils, the bacteria might have a high risk to be strongly recognized and eventually eliminated by the innate immune system. Structural and experimental studies showed that especially the 4´-phosphate group of lipid A is important for TLR4-MD2 recognition [10, 37]. We therefore hypothesized that under these conditions *H*. *pylori* might use ANX binding in the host to shield its phosphorylated lipid A molecules against the recognition by the innate TLR4 receptor expressed on neutrophils, macrophages or dendritic cells resident in the stomach mucosa. Indeed, we could show that *H*. *pylori* mutant strains producing phosphorylated lipid A (*lpxE/F* mutants) are significantly protected from TLR4 recognition when they were incubated with ANXA5, suggesting a role of ANX binding for an immune escape mechanism (Fig 5A). These results were corroborated by a strongly induced ANXA5 binding capacity and a significant reduction of TLR4-mediated NF-kB activation of the ANXA5-treated mouse colonizing *H*. *pylori* strain PMSS1 after mouse infection, as compared to the same strain grown for several passages *in vitro* (Fig 5C–5E).

Recently, the interaction of *H*. *pylori* with human conventional dendritic cells (cDCs) such as CD1c^+^ cDCs (cDC2s), which are among the first immune cells to encounter *H*. *pylori* in the gastric lining, has been studied [46]. These innate immune cells express the TLR4 receptor, which upon interaction with *H*. *pylori* drives the secretion of inflammatory Th1/Th17 cytokines (IL-1β, IL-12, IL-18 and IFN gamma) to shape a chemokine milieu for an effective immune response for bacterial clearance. Antibody-mediated blocking of TLR4 signaling in a mouse model resulted in down regulation of MyD88 expression, NF-kB activation and an increase in the numbers of CD4+CD25+Foxp3+Tregs, which increased the *H*. *pylori* colonization density [47]. Thus, our finding that the *H*. *pylori*-ANX interaction efficiently blocks TLR4 signaling might contribute to the bacterial strategy to efficiently subvert host cell clearance mechanisms.

CagA, the major pathogenicity factor of *H*. *pylori*, is injected into the host cell via the *cag*-T4SS, where it causes various effects [4]. We show here that binding of ANXA5 to *H*. *pylori* reduces the CagA translocation by up to ~33%, which is consistent with the data from Murata-Kamiya *et al*., who previously described that ANXA5 can reduce the CagA translocation by approximately 20% [38] (Fig 6A and 6B). They hypothesized that the reduced translocation is due to the blocking of phosphatidylserine (PS), which is involved in binding of CagA before translocation, since an anti-PS antibody showed the same effect. This does not exclude a possible direct interaction between ANXA5 and *H*. *pylori*, as described here. Thus, not only the host cell, but also the bacteria is a target for ANXA5 binding to modulate the efficiency of the *cag*-T4SS and translocation of CagA. A reduction of the CagA translocation as a consequence of a reduced binding of bacteria to the host cells was ruled out (S6A and S6B Fig).

In conclusion, our data clearly expand the functions of ANXs. In addition to their role as modulators of eukaryotic membrane trafficking and phagocytosis, novel functions for ANXs emerge as tools exploited by bacterial pathogens such as *H*. *pylori* to actively modulate its surface and interfere with recognition by the host immune system.

## Materials and methods

### Ethics statement

The study was approved by the Ethics Committee of the Nigerian Institute of Medical Research (IORG0002656) and of the Ludwig-Maximilians-Universität München (Registration number 335–08). All participants provided informed written consent.

#### Bacterial strains and cell line maintenance

*H*. *pylori* strains were grown on GC agar plates (Oxoid) supplemented with vitamin mix (1%) and horse serum (8%) (serum plates) or cholesterol (cholesterol plates) and cultured for 16 to 48 h in a microaerobic atmosphere (85% N_2_, 10% CO_2_, 5%O_2_) at 37°C. *E*. *coli* strains were grown on Luria–Bertani (LB) agar plates supplemented with antibiotics, as appropriate. Plasmids were introduced into *H*. *pylori* by transformation as described previously [48], and transformants were selected on serum plates containing 6 mg/l chloramphenicol, 8 mg/l kanamycin, 10 mg/l erythromycin, or 250 mg/l streptomycin, as appropriate. For liquid culture plate-grown *H*. *pylori* were resuspended in PBS and added in an OD of 0.075 into 6-well plates prepared with 2 ml Brucella Broth and 8 μl cholesterol (Gibco) as previously published [49]. After 24h at 37°C in a 10% CO_2_-atmosphere vitality was checked under the microscope before use. All cell lines were maintained at 37°C and 5% CO_2_ with the appropriate media. Generally, cells were grown in 75 cm^2^ tissue culture flasks (BD Falcon) and in 6-, 12- and 24-well plates (tissue culture treated cell culture clusters, Costar, Corning Inc.).

#### *H*. *pylori* mutant strain construction

*H*. *pylori* LPS mutants G27ΔHP1284, G27Δ*waaL*, 26695ΔHP1284 were generated as described earlier [31]. P12Δ*rfaE*, *G27*Δ*rfaE and 26695*Δ*rfaE* were constructed by transformation of the wt strains with plasmid pCL1. Therefore, a pSMART-hcKan vector harboring bp 908247–910341 of the P12 chromosome (= HP2kb04_M12) was amplified by inverse PCR using primers CL1 (ACG TGT CGA CTA GGA TTT TTT TCA TGC AC, *Sal*I) and CL2 (CAG GAT CCA AAA TTA AAA GGA CAC ATA ATG ATT GAT G, *Bam*HI) to generate the flanking regions for homologous recombination and ligated with a chloramphenicol resistance cassette (*Sal*I/*Bam*HI). Successful deletion mutants were confirmed by PCR using primers CL23 (ACA ATC AAA GCC ATA TCG CT) and CL24 (CCG CTG TTT CTG ATA CGA CCA).

*H*. *pylori* mutants in *lpxE*, *lpxF* and *eptA* genes were constructed by transformation of the wt strains with plasmids pBAS101, pBAS102, and pBAS103, respectively. For construction of these plasmids, a pSMART-hcKan vector containing bp 20052–22245, bp 1664943–1667251, or bp 20022–23512 of the P12 chromosome (= HP2kb07_L02 (*lpxE*)/HP2kb08_K01 *(lpxF*)/HP5kb04_C19 (*eptA*)) was amplified by inverse PCR using primer BAS201 (TAG GAT CCA AAC TTA ATT AAA AAA ACT TAA TTA AAG CTT TAA TTC, *Bam*HI) and BAS202 (TAG TCG ACT AAT TTT TTC ATG AGT GTT ATT TTA CTC TTT TTT G, *Sal*I) for *lpxE* deletion, BAS203 (TAG GAT CCT ATC AAT GGT AAA GGG ATA AAG TGC, *Bam*HI) and BAS204 (TAG TCG ACT CAT TGA AAC GCT CGC TTT TC, *Sal*I) for *lpxF* deletion, and BAS205 (TAG GAT CCC ACA AAA AAG AGT AAA ATA ACA CTC ATG, *Bam*HI) and BAS206 (TAG TCG ACG AAT AAT GAT GCC AAA CAC GC, *Sal*I) for *eptA* deletion. The product of BAS201/BAS202 was ligated with an *rpsL*^*S*^*erm*^*R*^ (*Sal*I/*Bam*HI) cassette for *lpxE* deletion, the products of BAS203/BAS204 and BAS205/206 were ligated with a chloramphenicol resistance cassette (*Sal*I/*Bam*H) for *lpxF* and *eptA* deletion, respectively. For confirmation of successful deletion, PCRs with primers BAS207 (TAG AGC CTG GTG AAG CCA TAG) and BAS208 (GCA ATT CTT TGG GAA AAA CAA ACG) for *lpxE* and *eptA* and BAS209 (GAC CTG TTG GGT GAA AGA GC) and BAS210 (CAA TAT TCA ATC CAA AAC GCA TGG) for *lpxF* were performed. Successful *lpxE* or *eptA* deletion mutants in G27 were confirmed using primers BAS207 and BAS211 (GCA ATT CTT TGG GGA AAA GAA ATG).

#### Infection with *H*. *pylori*

Before infection, AGS cells were synchronized by serum deprivation overnight. 1 h prior to infection, the medium was changed back to complete medium (CM). Cells enter the G_0_ quiescence when they grow in medium without serum. When the medium is again complemented with serum the cells ideally all enter the early G_1_ phase simultaneously [50]. Cells were infected with an MOI of 60 and incubated at 37°C, 5% CO_2_ for 1–3 h. 1 h prior to infection, ANXA5 was added to the *H*. *pylori* pre-culture. Alternatively, ANXA5 was added simultaneously at infection. To harvest the cells, they were scraped off in PBS supplemented with protease inhibitors (1 mM PMSF, 1 mM sodium vanadate, 1 μM leupeptin, 1 μM pepstatin). To prepare the samples for Western blotting, the pellet was resuspended in 20 μl PBS/protease inhibitors and 30 μl of 2X SDS loading buffer and boiled for 10 min.

#### *H*. *pylori* gastric colonization and re-isolation

All experiments and procedures were conducted in accordance with the Guidelines for the Care and Use of Laboratory Animals and approved by the Regierung von Oberbayern (ROB-55.2-2532.Vet_02-18-189). The PMSS1 strain was cultured on GC agar plates from frozen stocks (with 1% vitamin mix and 8% horse serum) for three days under microaerophilic condition (85% N_2_, 10% CO_2_, 5% O_2_). Mice were infected using an oral gavage needle for three consecutive days using approximately 6 x 10^7^ CFUs in Brucella Broth. 3 weeks post infection, *H*. *pylori* was re-isolated from the antrum and corpus following tissue homogenization using Wheaton homogenizations and selected on DENT plates (Oxoid *H*. *pylori* selective supplement–SR0147E containing Vancomycin, Cefsulodin Trimethoprim, Amphotericin B with Polymyxin B (Simga: P9602), Nalidixin acid (Roche), Bacitracin and 1% vitamin mix and 8% horse serum).

#### Protein separation by polyacrylamide gel and blotting

An adapted 6% single gel system based on Ahn et al [51] containing 0.01% trichloroethanol was used to separate the proteins based on their molecular weight, as previously published [52]. For a detailed protocol see https://dx.doi.org/10.17504/protocols.io.gipbudn). As a molecular weight marker, the Precision Plus Protein Allblue standards (BioRad) was used. The total number of proteins was detected in the ChemiDoc MP System (BioRad) using the stain-free technology [53]. In a semi-dry blotting chamber, the proteins were transferred to a PVDF membrane at a current of 1 mA/cm^2^ for 90 min. After blotting, the membrane was dried for a minimum of 1 h at 37°C or overnight at RT. The dry membrane with the proteins was re-activated by methanol. Blocking of unspecific binding sites was performed with TBS/0.075% Tween-20/3% bovine serum albumin (BSA) for 1 h at RT. The primary antibody was added and incubated for 1 h at room temperature (RT). After washing four times in TBS/0.075% Tween-20, the secondary antibodies were incubated for 45 min. at RT. For detection of horse radish peroxidase (HRP)-coupled secondary antibodies, the Millipore Immobilon Western solution was used. The signal was detected in the ChemiDoc MP System. For detection of phosphorylated CagA, the α-phosphotyrosine antibody (4G10) was added (1:10,000). Quantification of protein bands for CagA was performed using ImageLab software (Version 6.0.1, BioRad) and normalized to the stain-free gel. Alternatively, detection of fluorophore-coupled proteins was performed by exciting and detecting the fluorophore in the Gel Doc documentation system.

#### ANX binding assay

Bacteria were resuspended in PBS and incubated at an OD_550_ of 0.1 in an Eppendorf tube with 1 ml RPMI 1640 (Life Technologies) and 2.5 μl of ANXA1, ANXA2 or ANXA5. ANXA5 coupled with Alexa Fluor 647, 594 or 488 (Thermo Fisher). After 1 h incubation at 37°C in 10% CO_2_, the samples were prepared for further analysis by flow cytometry, Western blot or confocal microscopy. For flow cytometry analysis the samples were washed three times in ANXA5 binding buffer modified after Kenis et al., (10 mM HEPES, 150 mM NaCl, 5 mM KCl, 5 mM MgCl_2_, 1.8 mM CaCl_2_, pH 7.4) [30] with centrifugation at 4,000 rpm at 4°C for 5–10 min. The bacteria were resuspended in ANXA5 binding buffer and ANXA5 binding was measured as the increase of fluorescence in the 647-channel using the BD FACS Canto II. Flow cytometry data was analyzed using FlowJo software (version 7.6.1 or 10). Gates were set using the negative control (~ 2.5% of events in the positive gate). For statistical evaluation, the values of the negative controls (percentage and median FI) were subtracted from the obtained values of the other samples. For analysis with Western blot the samples were washed twice in ANXA5 binding buffer with centrifugation at 4,000 rpm at 4°C for 5–10 min and were then resuspended in 5 μl of PBS/Protease inhibitors and 7 μl of 2x SDS loading buffer.

#### Dot blot assay

LPS samples were diluted in PBS and applied to a PVDF membrane. Unspecific binding sites were blocked with TBS/0.05% Tween-20/5 mM CaCl_2_/3% BSA for 1 h at RT. All washing and incubation steps were performed with TBS/0.05% Tween-20/5 mM CaCl_2_. To detect ANXA5 binding, membranes were incubated with 2 μg/ml ANXA5 (Sigma, A9460) at 4°C overnight. After washing three times, the primary antibody (S3 Table) was incubated for 4 h at RT to detect ANXA5 binding. For detection of Lewis Y or lipid A (S3 Table), membranes were incubated with the respective primary antibodies at 4°C overnight. Blots were incubated with HRP-coupled secondary antibodies for 1 h at RT and detection was performed using Immobilon Chemiluminescent HRP Substrate (Millipore).

#### *H*. *pylori* binding assay

AGS cells were infected with GFP-expressing *H*. *pylori* at an MOI of 60. Bacteria were either pre-incubated with ANXA5 or ANXA5 was added simultaneously with the bacteria to the cells. After 1 h of incubation, the cells were put on ice, washed twice with ANXA5 binding buffer and harvested with a cell scraper directly before measurement in the flow cytometer (BD FACS Canto II). The GFP-signal was measured and used to determine the amount of bacteria that had bound to the cells.

#### LPS purification

LPS from *H*. *pylori* G27 was purified using the EDTA-promoted ultracentrifugation method as previously described [31]. Briefly, the bacterial cells harvested from 25 serum plates (24 h growth) were suspended into 15 ml of 10 mM Tris-HCl buffer (pH 8.0) containing 2 mM MgCl_2_. The bacterial cells in the suspension were disrupted by sonication and treated with DNase I and RNase A for 4 h in a 37°C water bath. Subsequently, 5 ml 0.5 M EDTA, 2.5 ml 20% SDS, and 2.5 ml 10 mM Tris-HCl buffer (pH 8.0) were added, and the pH was adjusted to 9.5. After centrifugation at 50,000 g for 30 min, the supernatant was transferred into a new tube and treated with proteinase K overnight in a 55°C water bath. After the addition of 2 volumes of 0.375 M MgCl_2_ in 95% ethanol, the mixed solution was centrifuged at 12,000 g for 15 min at 4°C, and the obtained LPS pellet was resuspended in 10 ml of 10 mM Tris-HCl (pH 8.0) containing 25 mM MgCl_2_. The solution was then ultracentrifuged at 100,000 g for 12 h at 15°C to obtain the purified LPS pellet, which was resuspended in H_2_O and lyophilized.

#### LPS microextraction for silver staining

LPS microextraction was prepared as described previously [54]. Briefly, bacterial cells (OD_550_ = 3.0) were harvested from serum plates and suspended in 100 μl LPS lysis buffer (2% SDS, 4% β-mercaptoethanol, 0.1% bromophenol blue, 10% glycerol, 1 M Tris-HCl (pH 6.8)). After heating the samples to 100°C for 10 min, 5 μl of proteinase K (20 mg/ml) were added to the cooled samples and incubated at 55°C over night. The samples were separated on a 15% SDS-PAGE and visualized by silver staining as described previously [55]. Briefly, LPS was oxidized with 40% ethanol, 5% acetic acid and 0.7% (w/v) periodic acid for 20 min at RT while shaking. After washing, the gel was stained (20 mM NaOH, 1.3% (v/v) ammonium hydroxide (29.4%), 0.7% (w/v) silver nitrate) for 10 min at RT while shaking. The gel was washed thoroughly and developed with 0.005% (w/v) citric acid monohydrate and 0.05% (v/v) formaldehyde (37%) for up to 10 min. The reaction was stopped with 10% acetic acid.

#### Confocal microscopy to study *H*. *pylori*–ANX interaction

After incubation of the bacteria with ANXA5 as described for the ANX binding assay, samples were fixed with 2.5% PFA for 15 min at RT and stained with DAPI (5 μg/ml) for 10 min at RT. Samples were settled in a 12 mm rounded cover slips laid in 24 wells plates by centrifugation at 3.000 rpm (Heraeus Megafuge 3.0) for 20 min at 4°C. The samples were mounted with mounting media on an object slide. Microscopy samples were examined with a Leica TCS SP5 confocal microscope, or Leica TCS SP8 confocal microscope and STED technology. Routine image processing was done with ImageJ 1.45h (National Institutes of Health) while Volocity 6.0.1 (Perkin Elmer) was used for co-localization analysis.

#### Immunohistochemistry of tissue slides and analysis by high resolution confocal microscopy

Paraffin sections were de-paraffinized and treated with sodium citrate buffer (10 mM sodium citrate, 0.05% Tween-20, pH 6.0) for antigen retrieval (microwave). Blocking was performed with 1% BSA, 2.5% goat serum and 0.5% saponin in PBS overnight at 4°C. Primary antibodies were added 1:50 (ANX) or 1:1000 (*H*. *pylori)* (S3 Table) in PBS/1% BSA/2.5% goat serum/0.5% saponin and samples were incubated overnight at 4°C. After washing, secondary antibodies (1:1000 in PBS/0.05% Tween-20/2.5% goat serum) were applied for 1 h at RT in the dark. Sections were stained with DAPI (5 μg/ml) for 10 min at RT in the dark and after washing were embedded in Fluorescence Mounting Medium (Dako). Sections were visualized using the Leica TCS SP5 confocal microscope for quantification of ANX signal or the confocal laser scanning microscope LSM880 (Zeiss) with Airyscan Module for co-localization studies. Imaris Bitmap 8.7 was used for co-localization analysis and Imaris software was used for the 3D reconstruction and rendering images.

#### Quantitative analysis of immunohistochemistry data

To analyze the stained sections, FIJI software (https://pubmed.ncbi.nlm.nih.gov/22743772) was used. For this, two macros were written, one counting the nuclei on each image and one measuring the fluorescence intensity of the stained protein of interest. The threshold for measuring the fluorescence intensity was chosen, so that no signal was detected in the isotype control samples. Fluorescence intensity was normalized to the number of nuclei on the respective image.

#### IL-8 secretion

The production of IL-8 by AGS cells after infection with *H*. *pylori* strains for 4 h was determined from cell supernatants by a sandwich enzyme linked immunosorbent assay (ELISA) (R&D Systems, USA), according to the manufacturer’s instructions, as previously described [56].

#### TLR4 assay

HEK-Blue hTLR-4 cells were obtained from Invivogen. Cells were cultured in DMEM/10% FCS/1% glutamine (Life Technologies) and HEK-Blue selection antibiotics (Invivogen) and sub-cultured every two to three days. Cells were seeded in a clear flat bottom 96-well plate (BD Falcon) one to two days prior to infection without selection antibiotics. Bacterial strains were incubated at an OD_550_ of 0.075 in 500 μl of F12 (Sigma)/10% FCS/5 mM CaCl_2_ for 2 h at 37°C and 10% CO_2_ while shaking. 1 h prior to infection, 1, 2, or 5 μg/ml ANXA5 (origene) were added to the bacteria. Cells were infected with an MOI of 100 for 20–24 h at 37°C and 5% CO_2_. QuantiBlue (Invivogen) was used for quantification according to the manufacturer’s instructions. After 30–120 min, the OD was measured at 655 nm using the Clariostar plate reader (BMG lab tech). As a positive control, cells were also incubated with 1 μg/ml of *E*. *coli* LPS (K-235, Sigma). For analysis, the value of mock infected cells was subtracted from all other values. Samples were measured in duplicates.

#### HiBiT assay

AGS [LgBiT] cells were seeded in a black, clear bottom 96-well plate (4titude) one to two days prior to infection without selection antibiotic. Bacteria, expressing HiBiT-CagA, were pre-incubated at an OD_550_ of 0.019 in 500 μl of F12 (Sigma)/5% FCS/5 mM CaCl_2_ for 2 h at 37°C, 10% CO_2_ and 100 rpm. 1 h prior to infection, 2, 5 or 10 μg/ml ANXA5 (origene) were added to the respective cultures. The cells were infected with an MOI of 25 and incubated for 2.5 h at 37°C and 5% CO_2_. After discarding the supernatant, 50 μl of luciferase substrate (Promega; 0.5 μl substrate, 9.5 μl assay buffer, 40 μl medium) were added and the luminescence signal was measured immediately using the Clariostar plate reader (BMG lab tech) as described previously.

#### Human biopsy samples

Gastric biopsy material was obtained from a gastrointestinal endoscopy study in Nigeria, which was supported by the Africa Infectiology Initiative of the Deutsche Forschungsgemeinschaft (DFG). Written informed consent of the patient was obtained prior to enrolment, in agreement with the Helsinki Declaration [57].

#### Statistical analysis

All statistical analyses were performed using the Graph Pad Prism 5 software. When two samples/conditions were compared, an unpaired Student´s t-test was used. When more than two samples/conditions were tested, a one-way ANOVA statistical analysis was performed. Either Bonferroni’s multiple comparison test of all columns or Dunnett’s multiple comparison test against a control column was used as *post hoc* test. Statistical analysis of two or more groups was performed using two-way ANOVA with Bonferroni’s *post hoc* test. P-values < 0.05 were considered as significant.

## Supporting information

S1 Fig ANXA5 binding characteristics of different *H. pylori* wt strains. To assess ANXA5 binding of different *H. pylori* strains (type I and type II), bacteria were incubated with ANXA5-A647 (red line) or left untreated (grey) and subsequently analyzed by flow cytometry. A representative histogram is shown for each strain. Median FI of the ANXA5-positive group was determined as described.
(TIF)

S2 Fig ANXA5 binding characteristics of selected bacterial species. The respective bacteria were incubated for 1 h with ANXA5-A647 (red) and subsequently analyzed by flow cytometry. Equally treated bacteria without ANXA5-A647 incubation served as negative control (grey). Representative histograms are shown.
(TIF)

S3 Fig Effect of growth conditions, media supplements and cholesterol-α-glucosyltransferase (Cgt) on ANXA5 binding. **A, C, D**) *H*. *pylori* P12-GFP harvested from plate or grown in liquid culture was incubated for 1 h with ANXA5-A647 and subsequently ANXA5 binding was analyzed as described. Additionally, samples of bacteria grown on plate were spiked with liquid culture medium (Lc-medium) to exclude interference of the medium conditions with the experimental outcome. (**A**) The percentage of ANXA5 positive events was calculated from three independent experiments. Data shown are means and SEM. Statistics were performed using one-way ANOVA and subsequent Bonferroni’s multiple comparison test. (*** p<0.001) (**C, D**) A representative histogram for each condition is shown. **B, E-G**) *H*. *pylori* P12-GFP was cultivated on agar plates supplemented with horse serum, sheep blood or cholesterol and subsequently incubated for 1 h with ANXA5-A647. Binding was analyzed by flow cytometry as described. (**B**) The percentage of ANXA5 positive events was calculated from three independent experiments. Data shown are as means with SEM. Statistics were performed using one-way ANOVA and subsequent Bonferroni’s multiple comparison test. No significant differences were observed. (**E-G**) A representative histogram for each condition is shown. **H-K)** *H*. *pylori* P12-GFP or the isogenic Δ*cgt* deletion mutant were incubated with (red line) or without (grey) ANXA5-A647 and fluorescence was determined via flow cytometry as described above. A representative histogram for each strain is shown in (**H**) and (**I**). The percentage of ANXA5 positive events was calculated from three independent experiments and are depicted as mean with SEM and statistics were preformed using Student’s unpaired t-test. No significant difference was observed (p = 0.7581).
(TIF)

S4 Fig Characterization of LPS of *H*. *pylori* wt and mutant strains. LPS samples from *H*. *pylori* wt and mutants were analysed by SDS-PAGE and visualized by silver staining. (**A**) SDS-PAGE and silver stain showing LPS structure of G27 wt, Δ*waaL*, Δ*rfaE* and HP1284 deletion. (**B**) SDS-PAGE and silver stain of G27 wt, Δ*lpxE*, Δ*lpxF* Δ*lpxE/F* and Δ*eptA* mutant strains. (**C**) SDS-PAGE and silver stain showing LPS structure of P12 and 26695 wt, Δ*rfaE* and ΔHP1284 deletion mutants.
(TIF)

S5 Fig Characterization of lipid A as binding target for ANXA5. *H*. *pylori* G27 (**A, D**), P12 (**B**) and 26695 (**C, E**) and the indicated isogenic mutant strains were incubated for 1 h with ANXA5-A647 and subsequently binding was evaluated by flow cytometry. Equally treated bacteria without ANXA-A647 addition served as negative control. Representative histograms are shown.
(TIF)

S6 Fig Impact of ANXA5 binding on *H*. *pylori* adherence and IL-8 induction. (**A**) AGS cells were infected with P12-GFP and optionally, ANXA5 was added simultaneously. After 1 h of co-incubation, AGS cells were washed to remove unbound bacteria and subsequently 488-fluorescence intensity was recorded by flow cytometry. AGS cells only served as negative control. Data shown are mean values with SEM of at least three independent experiments. No significant differences were observed between the sample with or without addition of ANXA5. (**B**) Same experiment as in (A) but ANXA5 was added to the bacterial pre-culture 1 h prior to the start of infection. Data shown are mean values with SEM of at least three independent experiments. Again, no negative impact of ANXA5 on adhesion of *H*. *pylori* to the host cells was observed. Statistical analysis: One-way ANOVA and Tukey’s multiple comparison post test; n = 3–4. (**C/D**) As in described (A) and (B), AGS cells were infected with *H*. *pylori* P12-GFP and ANXA5 was added either simultaneously (**C**) or bacteria were pre-treated with ANXA5 (**D**). After 4 h of co-incubation, supernatants were harvested and IL-8 concentrations were measured by ELISA. Equally treated AGS cells only were analyzed to determine background IL-8 secretion. No impact of ANXA5 on the IL-8 induction potential of the bacteria was observed. Statistical analysis was performed with one-way ANOVA and Tukey’s multiple comparison post test; n = 3.
(TIF)

S7 Fig Control staining for confocal laser scanning microscopy. **A)** Gastric tissue of an *H*. *pylori* infected patient (N2-97-A1) was stained for *H*. *pylori* (magenta) and DAPI (blue). For ANXA5, only the secondary antibody (green) was applied to control for unspecific binding. Confocal image was taken as a z-stack and the three-dimensional projection was reconstructed. **B**) Confocal image of gastric tissue of an *H*. *pylori* infected patient (N2-97-A1). Section was stained with secondary antibodies only (goat anti-mouse IgG-Alexa-488 and goat anti-rabbit IgG-Alexa-555) and DAPI to control for unspecific binding.
(TIF)

S1 Table Bacterial strains used in this study. (DOCX)

S2 Table Statistical analysis of binding of different bacterial species shown in Fig 2E. One-way ANOVA (p< 0.0001) was performed. Subsequently, Dunnett’s multiple comparison post-test was performed against the control columns *H*. *pylori* P12wt (as positive control for ANXA5 binding) and *C*. *jejuni* (as negative control for ANXA5 binding). All bacteria except *N*. *gonorrhoeae* N356 showed a highly significant reduction of ANXA5 binding when compared to P12wt. Only *N*. *gonorrhoeae* (N356, N302 and N309), *S*. *aureus*, *S*. *pneumoniae* and *M*. *catarrhalis* (25238) showed a significant or highly significant difference to the negative control, *C*. *jejuni*, and therefore are considered to bind ANXA5.
(DOCX)

S3 Table Antibodies used in this study. (DOCX)

S1 Movie *H*. *pylori* in human gastric tissue imaged in diverse rotated angles, for the distribution of annexin on the membrane surface of *H*. *pylori*, as shown in Fig 7G. (AVI)
