# Supplementary figures and images for: Helicobacter pylori binds human Annexins via Lipopolysaccharide to interfere with Toll-like Receptor 4 signaling
Source: PLoS Pathog. 2022 Feb 17;18(2):e1010326. doi: 10.1371/journal.ppat.1010326 (PMC8890734; doi:10.1371/journal.ppat.1010326)

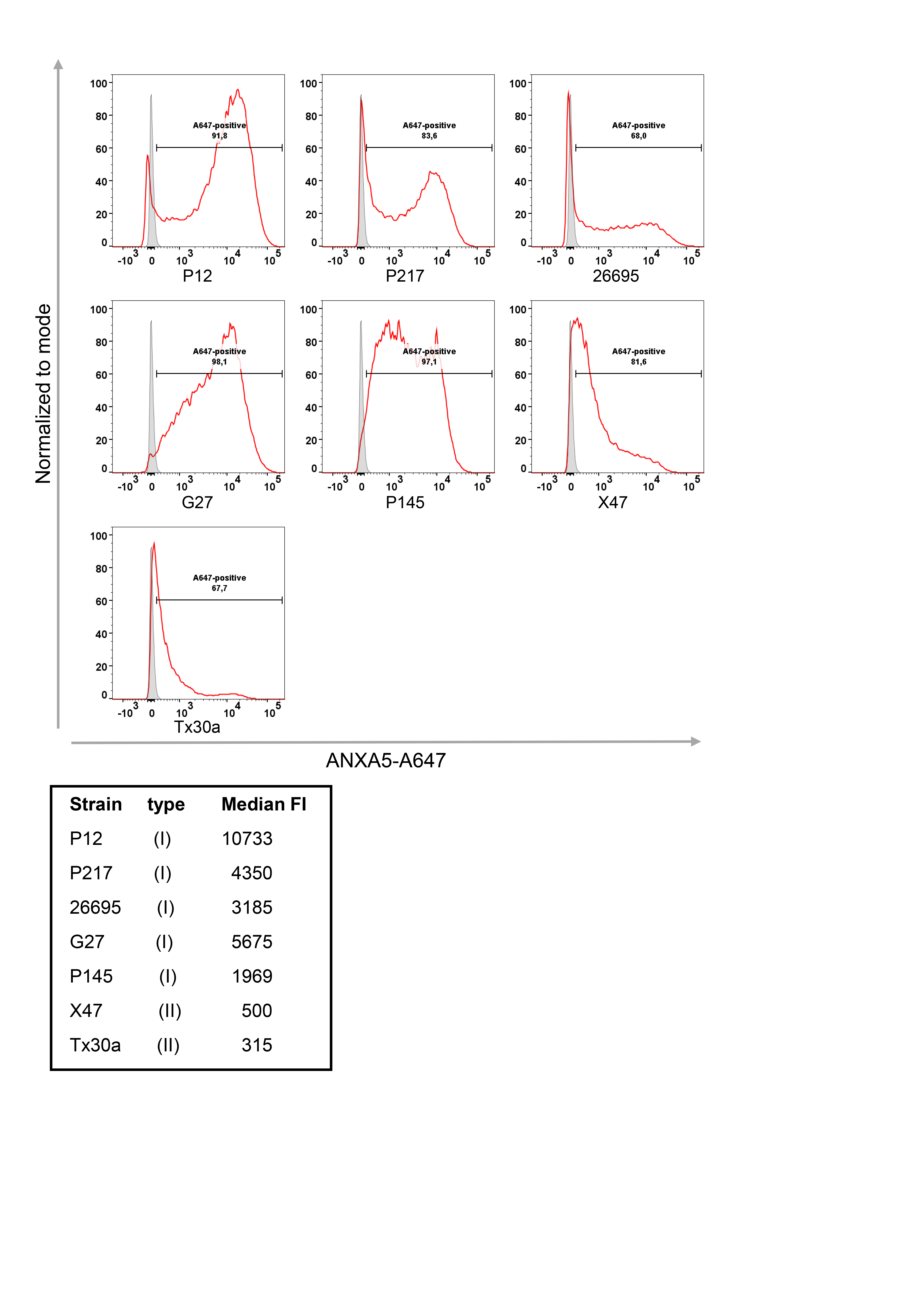

Supplement: S1 Fig — To assess ANXA5 binding of different H. pylori strains (type I and type II), bacteria were incubated with ANXA5-A647 (red line) or left untreated (grey) and subsequently analyzed by flow cytometry. A representative histogram is shown for each strain. Median FI of the ANXA5-positive group was determined as described. (TIF) [file ppat.1010326.s001.tif]

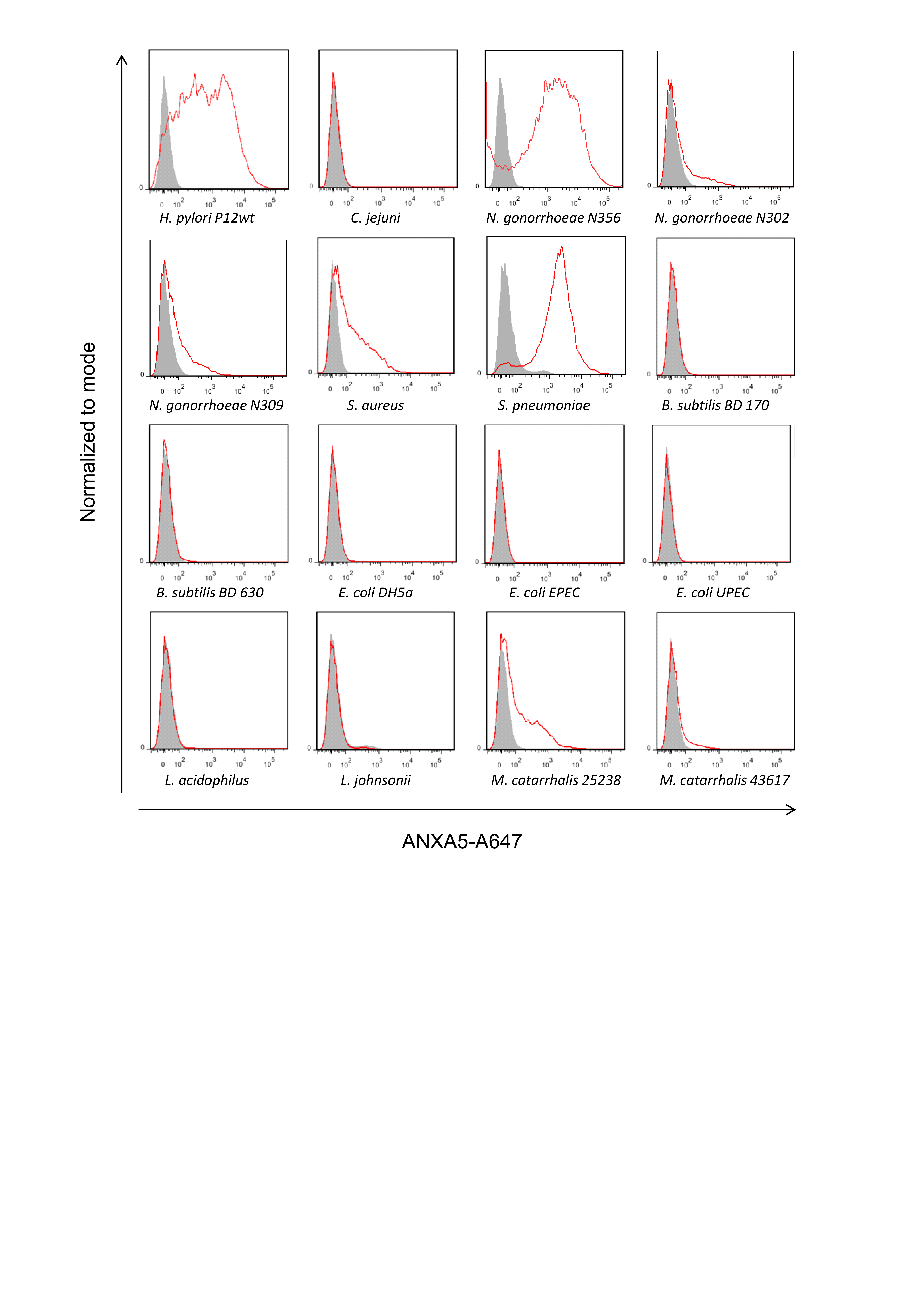

Supplement: S2 Fig — The respective bacteria were incubated for 1 h with ANXA5-A647 (red) and subsequently analyzed by flow cytometry. Equally treated bacteria without ANXA5-A647 incubation served as negative control (grey). Representative histograms are shown. (TIF) [file ppat.1010326.s002.tif]

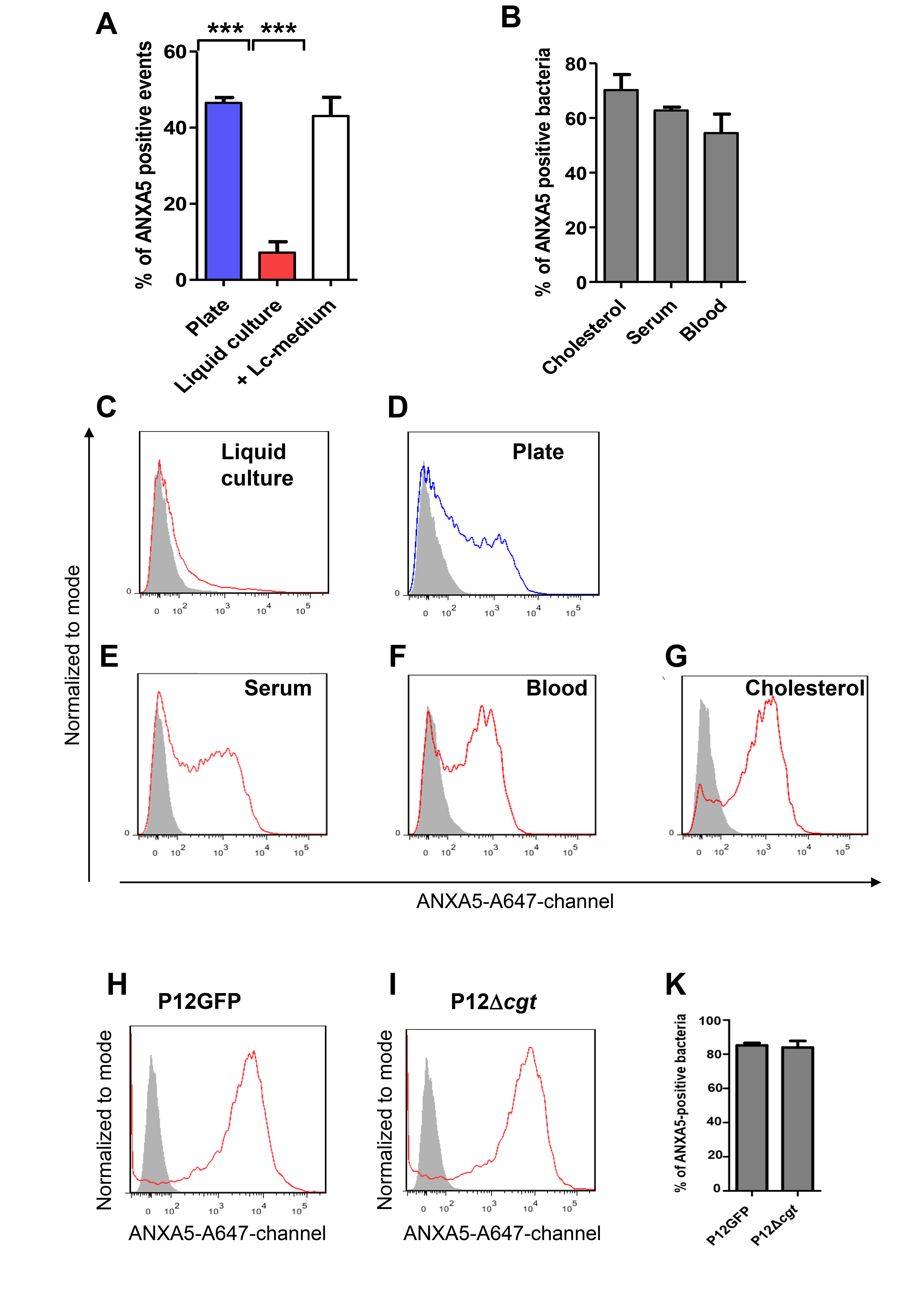

Supplement: S3 Fig — A, C, D) H. pylori P12-GFP harvested from plate or grown in liquid culture was incubated for 1 h with ANXA5-A647 and subsequently ANXA5 binding was analyzed as described. Additionally, samples of bacteria grown on plate were spiked with liquid culture medium (Lc-medium) to exclude interference of the medium conditions with the experimental outcome. (A) The percentage of ANXA5 positive events was calculated from three independent experiments. Data shown are means and SEM. Statistics were performed using one-way ANOVA and subsequent Bonferroni’s multiple comparison test. (*** p<0.001) (C, D) A representative histogram for each condition is shown. B, E-G) H. pylori P12-GFP was cultivated on agar plates supplemented with horse serum, sheep blood or cholesterol and subsequently incubated for 1 h with ANXA5-A647. Binding was analyzed by flow cytometry as described. (B) The percentage of ANXA5 positive events was calculated from three independent experiments. Data shown are as means with SEM. Statistics were performed using one-way ANOVA and subsequent Bonferroni’s multiple comparison test. No significant differences were observed. (E-G) A representative histogram for each condition is shown. H-K) H. pylori P12-GFP or the isogenic Δcgt deletion mutant were incubated with (red line) or without (grey) ANXA5-A647 and fluorescence was determined via flow cytometry as described above. A representative histogram for each strain is shown in (H) and (I). The percentage of ANXA5 positive events was calculated from three independent experiments and are depicted as mean with SEM and statistics were preformed using Student’s unpaired t-test. No significant difference was observed (p = 0.7581). (TIF) [file ppat.1010326.s003.tif]

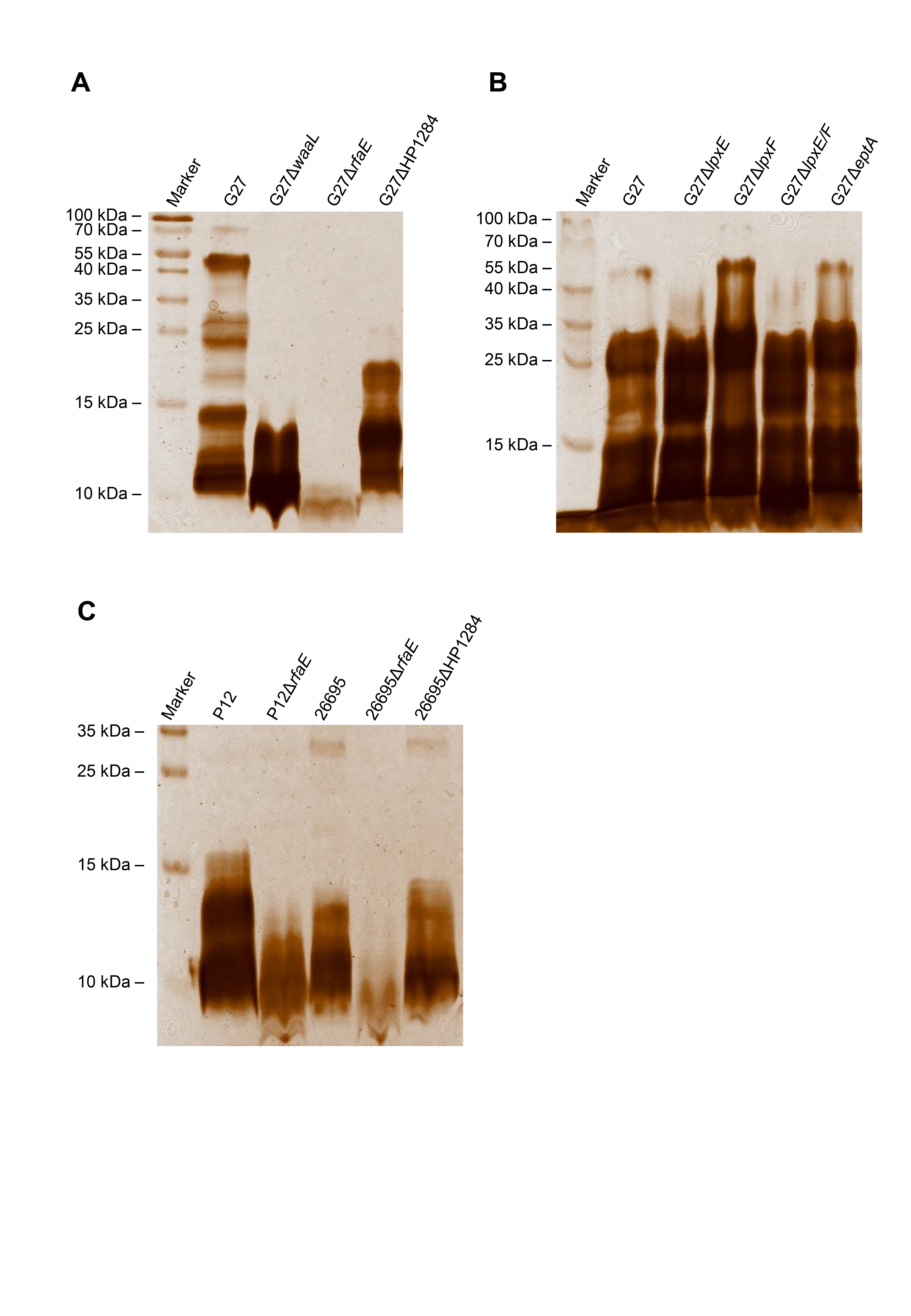

Supplement: S4 Fig — LPS samples from H. pylori wt and mutants were analysed by SDS-PAGE and visualized by silver staining. (A) SDS-PAGE and silver stain showing LPS structure of G27 wt, ΔwaaL, ΔrfaE and HP1284 deletion. (B) SDS-PAGE and silver stain of G27 wt, ΔlpxE, ΔlpxF ΔlpxE/F and ΔeptA mutant strains. (C) SDS-PAGE and silver stain showing LPS structure of P12 and 26695 wt, ΔrfaE and ΔHP1284 deletion mutants. (TIF) [file ppat.1010326.s004.tif]

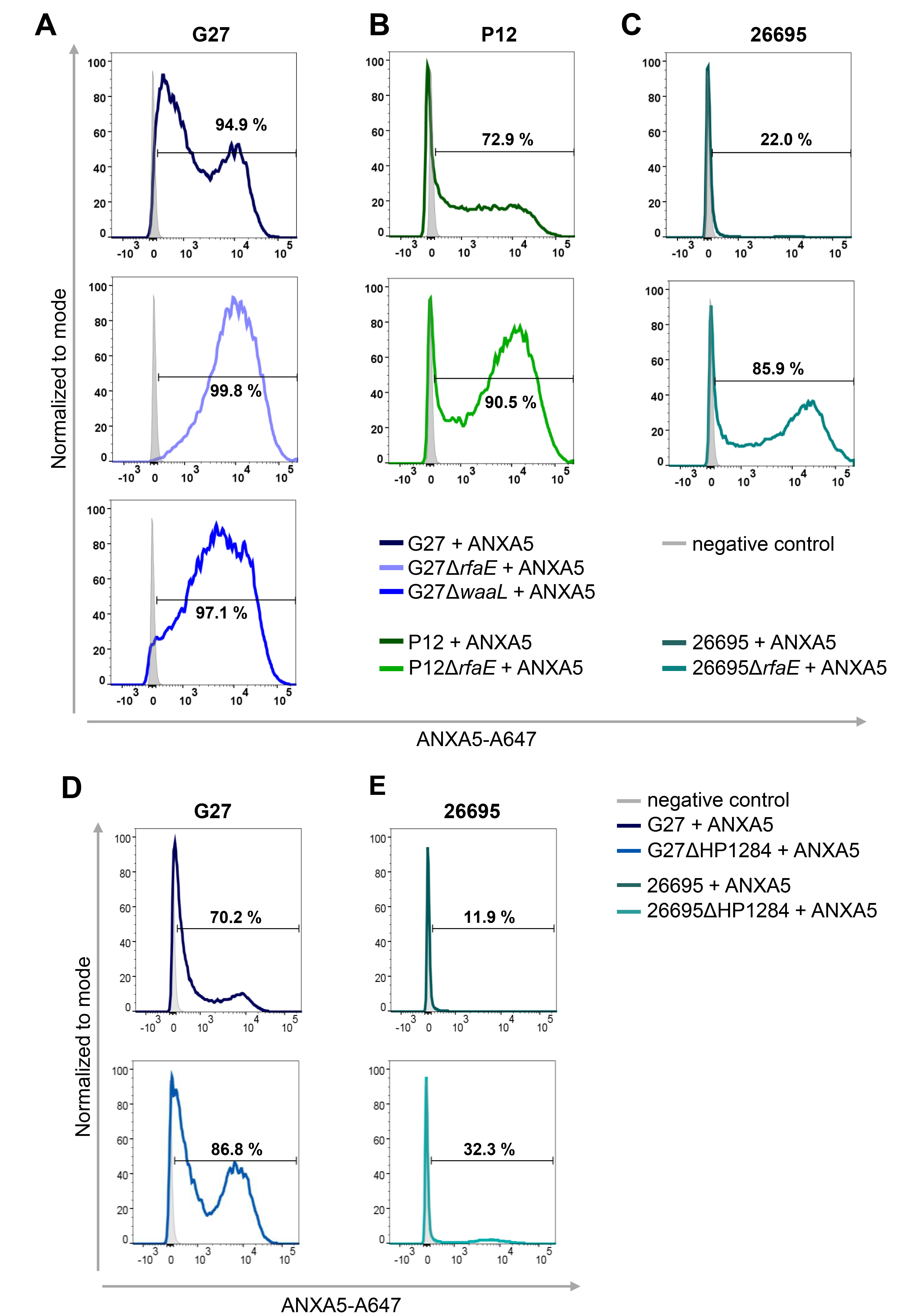

Supplement: S5 Fig — H. pylori G27 (A, D), P12 (B) and 26695 (C, E) and the indicated isogenic mutant strains were incubated for 1 h with ANXA5-A647 and subsequently binding was evaluated by flow cytometry. Equally treated bacteria without ANXA-A647 addition served as negative control. Representative histograms are shown. (TIF) [file ppat.1010326.s005.tif]

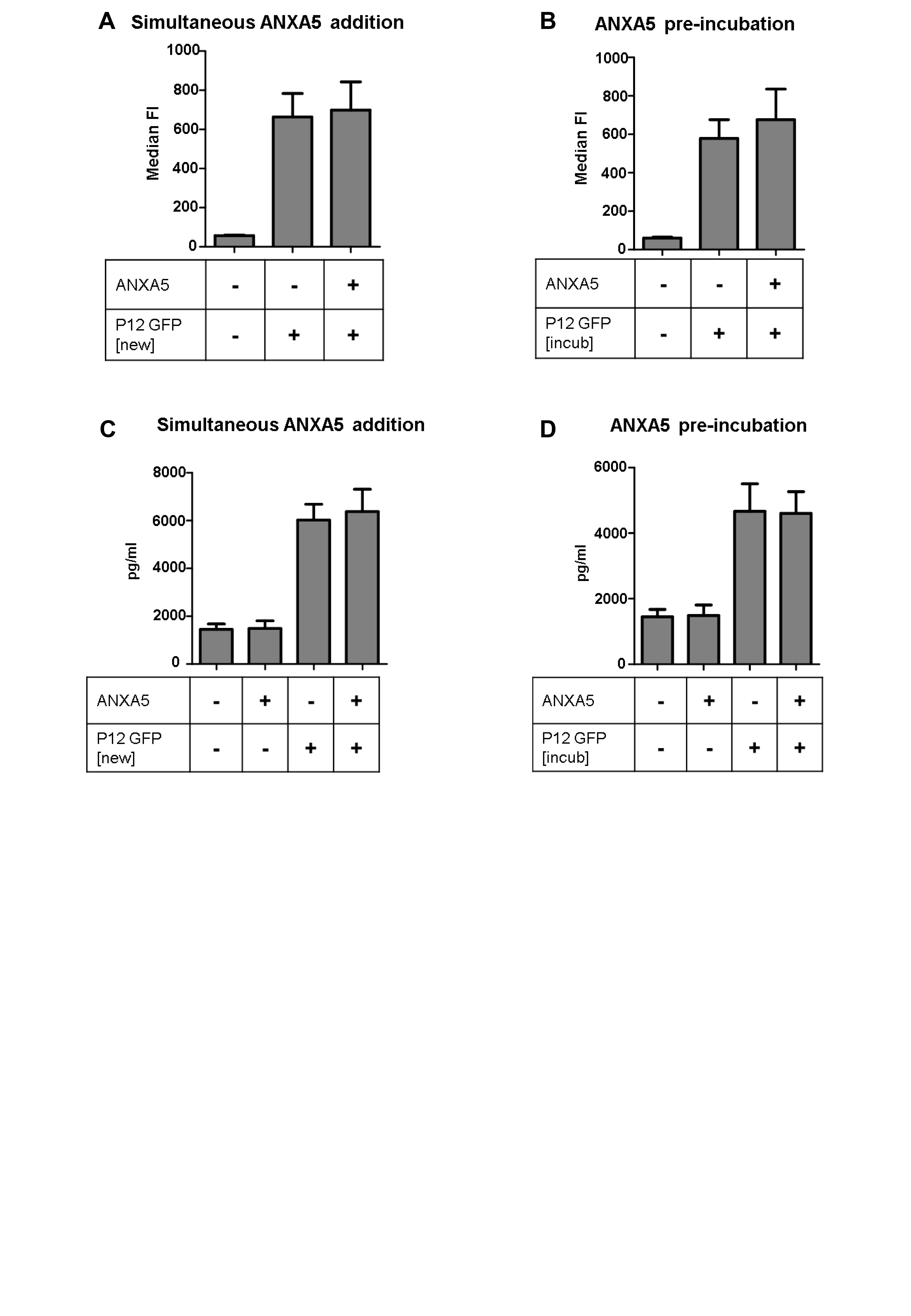

Supplement: S6 Fig — (A) AGS cells were infected with P12-GFP and optionally, ANXA5 was added simultaneously. After 1 h of co-incubation, AGS cells were washed to remove unbound bacteria and subsequently 488-fluorescence intensity was recorded by flow cytometry. AGS cells only served as negative control. Data shown are mean values with SEM of at least three independent experiments. No significant differences were observed between the sample with or without addition of ANXA5. (B) Same experiment as in (A) but ANXA5 was added to the bacterial pre-culture 1 h prior to the start of infection. Data shown are mean values with SEM of at least three independent experiments. Again, no negative impact of ANXA5 on adhesion of H. pylori to the host cells was observed. Statistical analysis: One-way ANOVA and Tukey’s multiple comparison post test; n = 3–4. (C/D) As in described (A) and (B), AGS cells were infected with H. pylori P12-GFP and ANXA5 was added either simultaneously (C) or bacteria were pre-treated with ANXA5 (D). After 4 h of co-incubation, supernatants were harvested and IL-8 concentrations were measured by ELISA. Equally treated AGS cells only were analyzed to determine background IL-8 secretion. No impact of ANXA5 on the IL-8 induction potential of the bacteria was observed. Statistical analysis was performed with one-way ANOVA and Tukey’s multiple comparison post test; n = 3. (TIF) [file ppat.1010326.s006.tif]

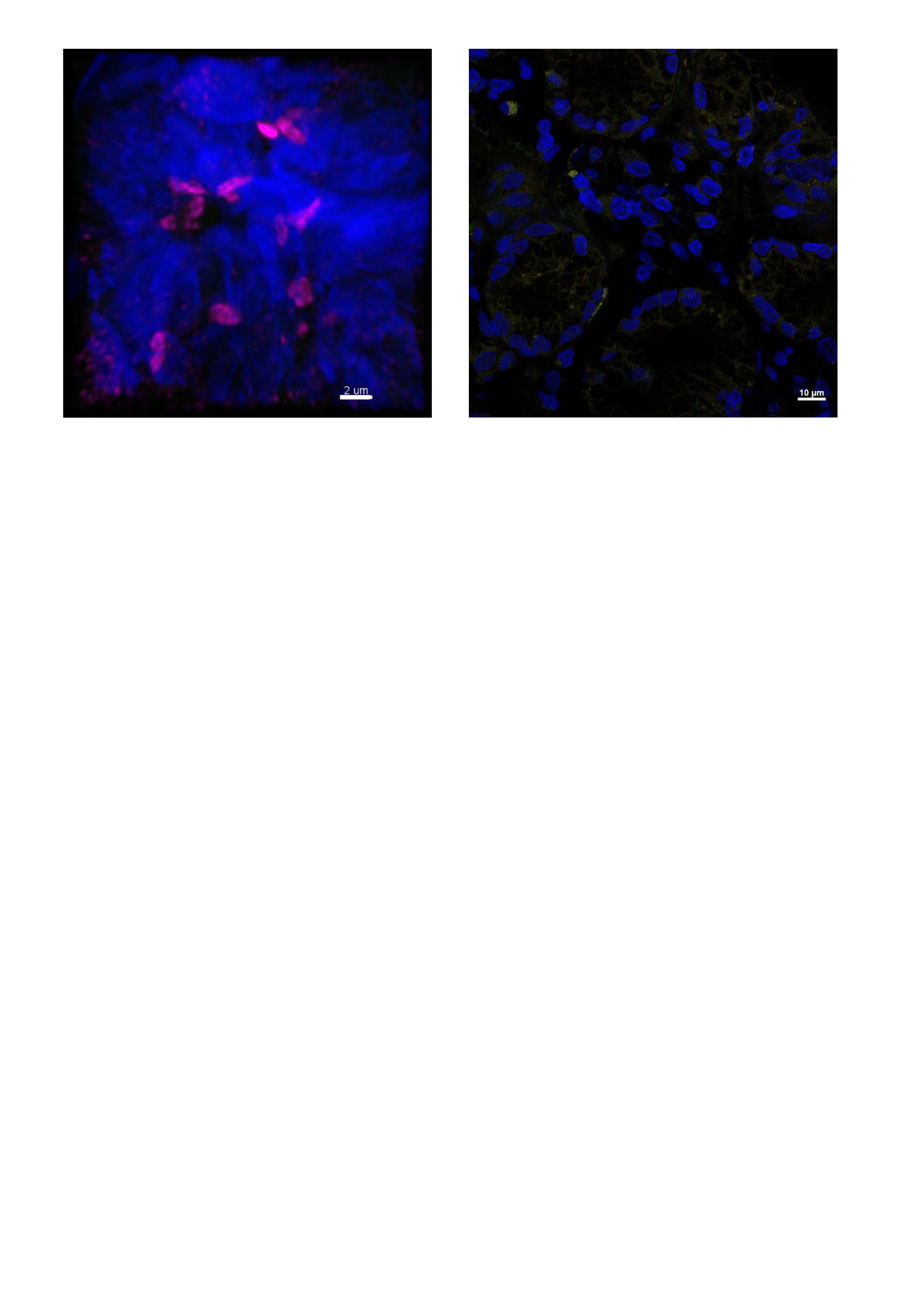

Supplement: S7 Fig — A) Gastric tissue of an H. pylori infected patient (N2-97-A1) was stained for H. pylori (magenta) and DAPI (blue). For ANXA5, only the secondary antibody (green) was applied to control for unspecific binding. Confocal image was taken as a z-stack and the three-dimensional projection was reconstructed. B) Confocal image of gastric tissue of an H. pylori infected patient (N2-97-A1). Section was stained with secondary antibodies only (goat anti-mouse IgG-Alexa-488 and goat anti-rabbit IgG-Alexa-555) and DAPI to control for unspecific binding. (TIF) [file ppat.1010326.s007.tif]
