# Supplementary material for: Helicobacter pylori binds human Annexins via Lipopolysaccharide to interfere with Toll-like Receptor 4 signaling
Source: PLoS Pathog. 2022 Feb 17;18(2):e1010326. doi: 10.1371/journal.ppat.1010326 (PMC8890734; doi:10.1371/journal.ppat.1010326)
Supplement: S1 Table — (DOCX) [file ppat.1010326.s008.docx]

**S1 Table: Bacterial strains used in this study**

| **Bacterial strains and mutants** | **Special features of strain** | **Reference** |
| --- | --- | --- |
| P12 | *H. pylori* wt strain | [1] |
| P12[HiBiT-CagA] | Reporter strain to determine CagA translocation | [2] |
| P12[HiBiT-CagA]Δ*cagT* | Deletion of *cagT* gene, defective T4SS | [2] |
| P12[HiBiT-CagA]Δ*rfaE* | Deletion of *rfaE* gene (HP0858) | this study |
| P12-GFP | Green fluorescent *H. pylori* strain | [3] |
| P12Δ*cgt* | Deletion of *cgt* gene | this study |
| P12*∆rfaE* | Deletion of *rfaE* gene (HP0858) | this study |
| P12*∆*HP1284 | Deletion of HP1284 gene | [4] |
| 26695 | wt *H. pylori* strain | [6] |
| 26695*∆rfaE* | Deletion of *rfaE* gene (HP0858) | this study |
| G27 | wt *H. pylori* strain | [7] |
| G27*∆waaL* | wt *H. pylori* strain with deletion of *waaL* gene | [4] |
| G27*∆rfaE* | Deletion of *rfaE* gene (HP0858) | this study |
| G27*∆*HP1284 | Deletion of HP1284 gene | [4] |
| G27*∆*HP0805 | Deletion of HP0805 gene | [4] |
| G27*∆lpxE* | Deletion of *lpxE* gene (HP0021) | this study |
| G27*∆lpxF* | Deletion of *lpxF* gene (HP1580) | this study |
| G27*∆lpxE/F* | Deletion of *lpxE* (HP0021) and *lpxF* gene (HP1580) | this study |
| G27*∆eptA* | Deletion of *eptA* gene (HP0022) | this study |
| G27*∆cag*PAI | Deletion of *cag*PAI | this study |
| G27*∆lpxE/F∆cag*PAI | Deletion of *lpxE* (HP0021) and *lpxF* gene (HP1580) and deletion of *cag*PAI | this study |
| PMSS1 | wt *H. pylori* strain, able to infect mice | [8] |
| X47 | wt *H. pylori* strain, able to infect mice | [9] |
| TX30a | wt *H. pylori* strain, type II strain, ATCC 51932 | [10] |
| P145 | wt *H. pylori* strain, ATCC 45526 | [11] |
| P217 | wt *H. pylori* strain | [11] |
| *M. catharralis* 25238 | *Moraxella catharralis* wt strain | [3] |
| *M. catharralis* 43617 | *Moraxella catharralis* wt strain | [3] |
| *N. gonorrhoeae* | *Neisseria gonorrhoeae* strains N302, 309, 3056, all MS11 strains | T.F. Meyer |
| *S. aureus* | *Staphylococcus aureus*, patient isolate | ATCC 29213 |
| *S. pneumoniae* | *Streptococcus pneumonia* patient isolate | ATCC 49619 |
| *B. subtilis* |  | this study |
| *E. coli* DH5α |  | lab strain |
| *E. coli* EPEC | Enteropathogenic *E. coli* isolate,  Max von Pettenkofer-Institute, LMU Munich | this study |
| *E. coli* UPEC | Uropathogenic *E. coli* isolate,  Max von Pettenkofer-Institute, LMU Munich | this study |
| *L. acidophilus* | *Lactobacillus acidophilus NCC-12* | Nestlé, Lausanne |
| *L. johnsonii* | *Lactobacillus johnsonii, NCC-1680* | Nestlé, Lausanne |

**References**

1. Schmitt W, Haas R. Genetic analysis of the *Helicobacter pylori* vacuolating cytotoxin: structural similarities with the IgA protease type of exported protein. Mol Microbiol. 1994;12:307-19.

2. Lettl C, Haas R, Fischer W. Kinetics of CagA type IV secretion by Helicobacter pylori and the requirement for substrate unfolding. Mol Microbiol. 2021. Epub 2021/06/15. doi: 10.1111/mmi.14772. PubMed PMID: 34121254.

3. Königer V, Holsten L, Harrison U, Busch B, Loell E, Zhao Q, et al. *Helicobacter pylori* exploits human CEACAMs via HopQ for adherence and translocation of CagA. Nat Microbiol. 2016;2:16188-99. doi: nmicrobiol2016188 [pii];10.1038/nmicrobiol.2016.188 [doi].

4. Li H, Yang T, Liao T, Debowski AW, Nilsson HO, Fulurija A, et al. The redefinition of *Helicobacter pylori* lipopolysaccharide O-antigen and core-oligosaccharide domains. PLoS Pathog. 2017;13(3):e1006280. doi: 10.1371/journal.ppat.1006280 [doi];PPATHOGENS-D-17-00156 [pii].

5. Schindele F, Weiss E, Haas R, Fischer W. Quantitative analysis of CagA type IV secretion by *Helicobacter pylori* reveals substrate recognition and translocation requirements. Mol Microbiol. 2016;100(1):188-203. doi: 10.1111/mmi.13309 [doi].

6. Tomb J-F, White O, Kerlavage AR, Clayton RA, Sutton GG, Fleischmann RD, et al. The complete genome sequence of the gastric pathogen *Helicobacter pylori*. Nature. 1997;388:539-47.

7. Baltrus DA, Amieva MR, Covacci A, Lowe TM, Merrell DS, Ottemann KM, et al. The complete genome sequence of *Helicobacter pylori* strain G27. J Bacteriol. 2009;191(1):447-8. doi: JB.01416-08 [pii];10.1128/JB.01416-08 [doi].

8. Arnold IC, Lee JY, Amieva MR, Roers A, Flavell RA, Sparwasser T, et al. Tolerance rather than immunity protects from *Helicobacter pylori*-induced gastric preneoplasia. Gastroenterology. 2011;140(1):199-209. doi: S0016-5085(10)00956-X [pii];10.1053/j.gastro.2010.06.047 [doi].

9. Kleanthous H, Tibbitts TJ, Gray HL, Myers GA, Lee CK, Ermak TH, et al. Sterilizing immunity against experimental *Helicobacter pylori* infection is challenge-strain dependent. Vaccine. 2001;19(32):4883-95.

10. Atherton JC, Cao P, Peek RM, Tummuru MKR, Blaser MJ, Cover TL. Mosaicism in vacuolating cytotoxin alleles of *Helicobacter pylori*. J Biol Chem. 1995;270:17771-7.

11. Jimenez-Soto LF, Kutter S, Sewald X, Ertl C, Weiss E, Kapp U, et al. *Helicobacter pylori* type IV secretion apparatus exploits beta1 integrin in a novel RGD-independent manner. PLoS Pathog. 2009;5(12):e1000684. doi: 10.1371/journal.ppat.1000684 [doi].
