# Supplementary material for: Helicobacter pylori binds human Annexins via Lipopolysaccharide to interfere with Toll-like Receptor 4 signaling
Source: PLoS Pathog. 2022 Feb 17;18(2):e1010326. doi: 10.1371/journal.ppat.1010326 (PMC8890734; doi:10.1371/journal.ppat.1010326)
Supplement: S2 Table — One-way ANOVA (p< 0.0001) was performed. Subsequently, Dunnett’s multiple comparison post-test was performed against the control columns H. pylori P12wt (as positive control for ANXA5 binding) and C. jejuni (as negative control for ANXA5 binding). All bacteria except N. gonorrhoeae N356 showed a highly significant reduction of ANXA5 binding when compared to P12wt. Only N. gonorrhoeae (N356, N302 and N309), S. aureus, S. pneumoniae and M. catarrhalis (25238) showed a significant or highly significant difference to the negative control, C. jejuni, and therefore are considered to bind ANXA5. (DOCX) [file ppat.1010326.s009.docx]

**S2 Table: Statistical analysis of binding of different bacterial species shown in Fig 2E.**

|  | **Compared to control** | | ANXA5  binding |
| --- | --- | --- | --- |
|  | Positive  (*H. pylori* P12) | Negative  (*C. jejuni*) |  |
| ***N. gonorrh.* N356** | n.s. | *** | yes |
| ***N. gonorrh.* N302** | *** | * | yes |
| ***N. gonorrh.* N309** | *** | * | yes |
| ***S. aureus*** | *** | *** | yes |
| ***S. pneumoniae*** | *** | *** | yes |
| ***B. subtilis* BD 170** | *** | n.s. | no |
| ***B. subtilis* BD 630** | *** | n.s. | no |
| ***E. coli* DH5α** | *** | n.s. | no |
| ***E. coli* EPEC** | *** | n.s. | no |
| ***E. coli* UPEC** | *** | n.s. | no |
| ***L. acidophilus*** | *** | n.s. | no |
| ***L. johnsonii*** | *** | n.s. | no |
| ***M. cat.* 25238** | *** | *** | yes |
| ***M. cat.* 43617** | *** | n.s. | no |
