# Supplementary material for: Helicobacter pylori binds human Annexins via Lipopolysaccharide to interfere with Toll-like Receptor 4 signaling
Source: PLoS Pathog. 2022 Feb 17;18(2):e1010326. doi: 10.1371/journal.ppat.1010326 (PMC8890734; doi:10.1371/journal.ppat.1010326)
Supplement: S3 Table — (DOCX) [file ppat.1010326.s010.docx]

**S3 Table: Antibodies used in this study**

| **Name** | **Target** | **Supplier** | **Application** |
| --- | --- | --- | --- |
| ab196830 | ANXA1 | abcam | IF |
| ERP13054(B) | ANXA2 | abcam | IF |
| 1F4-1A5 | ANXA5 | abcam | IF, Dot blot |
| AK175 | *H. pylori* | This lab | IF |
| PA1-73178 | Lipid A | Thermo Fisher | Dot blot |
| F3 | Lewis Y | abcam | Dot blot |
| 4G10 | Phospho-Tyrosine | Upstate Millipore | WB |
| AK299 | CagA | This lab | WB |
| AK263 | RecA | This lab | WB |
